# *bric à brac* controls sex pheromone choice by male European corn borer moths

**DOI:** 10.1038/s41467-021-23026-x

**Published:** 2021-05-14

**Authors:** Melanie Unbehend, Genevieve M. Kozak, Fotini Koutroumpa, Brad S. Coates, Teun Dekker, Astrid T. Groot, David G. Heckel, Erik B. Dopman

**Affiliations:** 1 Department of Entomology, Max Planck Institute for Chemical Ecology, Jena, Germany; 2 Department of Biology, Tufts University, Medford, MA USA; 3 Institute for Biodiversity and Ecosystem Dynamics, University of Amsterdam, Amsterdam, XH the Netherlands; 4 INRAE, Sorbonne Université, CNRS, IRD, UPEC, Université Paris Diderot, Institute of Ecology and Environmental Sciences of Paris, Versailles, Cedex France; 5 USDA-ARS, Corn Insects and Crop Genetics Research Unit, Ames, IA USA; 6 Department of Plant Protection Biology, Swedish University of Agricultural Sciences, Alnarp, Sweden; 7 Present Address: Department of Biology, University of Massachusetts Dartmouth, Dartmouth, MA USA

**Keywords:** Behavioural ecology, Evolutionary genetics, Speciation, Behavioural genetics

## Abstract

The sex pheromone system of ~160,000 moth species acts as a powerful form of assortative mating whereby females attract conspecific males with a species-specific blend of volatile compounds. Understanding how female pheromone production and male preference coevolve to produce this diversity requires knowledge of the genes underlying change in both traits. In the European corn borer moth, pheromone blend variation is controlled by two alleles of an autosomal fatty-acyl reductase gene expressed in the female pheromone gland (*pgFAR*). Here we show that asymmetric male preference is controlled by *cis*-acting variation in a sex-linked transcription factor expressed in the developing male antenna, *bric à brac* (*bab*). A genome-wide association study of preference using pheromone-trapped males implicates variation in the 293 kb *bab* intron 1, rather than the coding sequence. Linkage disequilibrium between *bab* intron 1 and *pgFAR* further validates *bab* as the preference locus, and demonstrates that the two genes interact to contribute to assortative mating. Thus, lack of physical linkage is not a constraint for coevolutionary divergence of female pheromone production and male behavioral response genes, in contrast to what is often predicted by evolutionary theory.

## Abstract (web summary)

Many organisms, including moths, use pheromones to attract mates. A study using multiple genomic tools and gene editing identifies a new, neuronal gene underlying mate preference and shows that signal and response loci are in linkage disequilibrium despite being physically unlinked.

## Introduction

Chemical communication influences mate choice in diverse forms of life, from fungi and arthropods to fishes and mammals^1,2^, and a large body of theory has developed to account for widespread shifts in signals and preferences^3–5^. Yet, empirical research needed to evaluate theoretical predictions for the divergence of sexual communication has lagged, in part, because change in signal-preference combination has rarely been linked to causal genes.

The European corn borer moth *Ostrinia nubilalis* (Hübner) (Lepidoptera: Crambidae) has been a model system for pheromone divergence ever since two different strains, E and Z, were discovered ~50 years ago^6^. Females of both strains produce a volatile pheromone consisting of (Z)-11-tetradecenyl acetate (Z11-14:OAc) and (E)-11-tetradecenyl acetate (E11-14:OAc), but in opposite ratios, 97:3 Z:E ratio in the Z-strain and 1:99 Z:E in the E-strain^6^. Blend differences are caused by allelic variation in the autosomal gene *pgFAR*, encoding a pheromone gland fatty-acyl reductase with strain-specific substrate specificity^7^.

Previous work determined that differential pheromone preference in males is encoded by a sex-linked response locus named *Resp* located on the Z chromosome^8–10^. A cluster of pheromone receptor loci expressed in male antennae^11,12^ are also sex-linked. These were considered candidates for *Resp* because an earlier study of the moths *Heliothis virescens* and *H. subflexa* showed genetic switching of species-specific pheromone receptors caused a switch of species-specific male preference^13^. However, *Ostrinia* pheromone receptors mapped about 20 cM away from *Resp* as determined by an AFLP linkage map^9,14^. This was confirmed by a quantitative trait locus (QTL) mapping study that more precisely located the pheromone receptor cluster 15 cM away^15^.

Here, we apply an approach that merges behavioral and electrophysiological phenotyping, expression profiling and gene editing, and genomic scans of assortative mating and associations with preference in nature, to identify *bric à brac* (*bab*) as the gene controlling mate choice in males. *bab*’s upregulation during early neuronal development in male pupal antennae and its slight temporal expression shift between strains are consistent with this role. Recombination breakpoints in introgression lines upstream of *bab* exon 2, a lack of fixed amino-acid substitutions within exon 1, and a nonessential role of a novel exon 1.5 after CRISPR knockout, all combine to rule out *bab* exons as determining male preference. Instead, a genome-wide association study (GWAS) of pheromone preference under field conditions indicates that preference is controlled by sequence variation within the 293 kb *bab* intron 1. Finally, although the pheromone production and response genes are located on different chromosomes in *Ostrinia* as they are in many other Lepidoptera^9^, strong genomic associations occur between coding changes at *pgFAR* alleles and specific polymorphisms within *bab* intron 1.

## Results

### QTL to candidate gene in lab populations

Previous QTL mapping identified *Resp* to an 8 cM region between the genes *terribly reduced optic lobes* (*trol*) and *CCR4-NOT* (*not*) on the Z chromosome^15^. To fine-map *Resp* we created recombinant inbred lines with single crossovers between *trol* and *not* (Supplementary Fig. 1). Phenotyping of males using behavioral attraction in a wind tunnel and electrophysiological recordings narrowed the interval containing *Resp* to a region consisting of six genes *Bap18*, *LIM homeobox*, *Bgi12353B*, *Bgi12353A*, *archipelago (ago)*, and *bric à brac* (*bab*) (Supplementary Fig. 2).

**Fig. 1:**
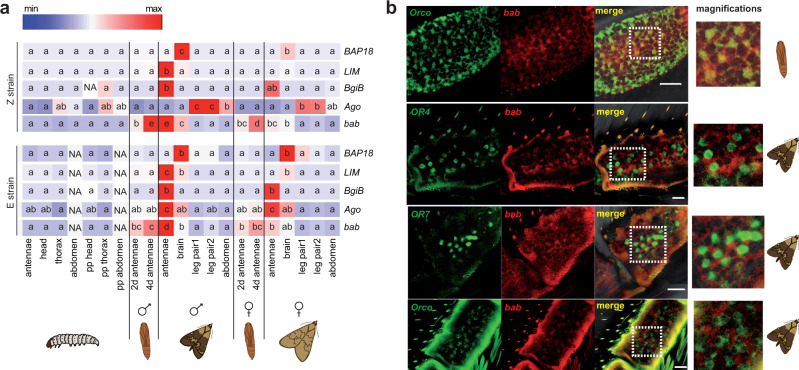
Tissue-specific and cell-specific gene expression. **a** Heat map showing tissue-specific, sex-specific, and strain-specific mean expression of *Bap18*, *LIM*, *BgiB ago*, and *bab* as determined by RT-qPCR. Red signifies higher expression; pp prepupal stage; d days. ANOVAs per candidate gene, and separately for each strain, yielded significant variation among all tissue by life-stage combinations, after a Benjamini–Hochberg multiple-test correction. Letters indicate significant differences in two-sided Tukey’s HSD post-hoc tests (*P* < 0.05). Z strain: *Bap18 F* = 44.83, df = 20, *P* = 4 × 10^−16^; *BgiB F* = 3.81, df = 20, *P* = 9.01 × 10^−5^; *LIM F* = 37.97, df = 20, *P* = 4 × 10^−16^; *ago F* = 52.11, df = 20, *P* = 4 × 10^−16^; *bab F* = 155.2, df = 20, *P* = 4 × 10^−16^; E strain: *Bap18 F* = 7.1, df = 18, *P* = 2.41 × 10^−07^; *BgiB F* = 7.13, df = 18, *P* = 1.26 × 10^−07^; *LIM F* = 181.8, df = 18, *P* = 4 × 10^−16^; *ago F* = 18.31, df = 18, *P* = 2.61 × 10^−13^; *bab F* = 26.29, df = 18, *P* = 7.35 × 10^−16^. **b** Double whole mount in situ hybridization of differentially labeled transcripts of *bab* in red (digoxigenin) and odorant receptors and co-receptor in green (biotin). Top images show cells co-expressing both *bab* and *Orco* in 4-day-old male pupal antenna. Bottom images show separate but neighboring cells expressing *bab* and *OR7*, *OR4*, or *Orco* (co-localization in the same sensillum) in 2-day-old adult male antenna. In merged images on the right, yellow indicates overlapping of the two signals. Successful demonstrations in pupal antennae were obtained 4 times for *bab*/*Orco* combinations. Successful demonstrations in adult antennae were obtained 22 times for *bab*/*OR4* combinations, 8 times for *bab*/*OR7* combinations and 7 times for *bab*/*Orco* combinations. The scale bar corresponds to 20 µm. Source data are provided as a Source Data file.

To evaluate these genes as candidates, we conducted quantitative PCR of Z- and E-strain laboratory populations across developmental stages and sexes (*n* = 3 biological replicates per tissue type). Across the 21 tissues studied, we specifically looked for pupal stage expression, since axonal connections developing from olfactory sensory neurons (OSNs) in the antenna to the antennal lobe in the brain are established in the pupa in Lepidoptera^16^, and newly emerged *O. nubilalis* adult males can detect female sex pheromone and readily mate^17^. Of five candidate genes with detectable levels of expression, *bab* was the only one significantly upregulated at the pupal stage (Fig. 1a). Compared to other instars and tissues, *bab* expression was highest in pupal and adult male antennae, but it was also moderately expressed in adult male brain, and to a lesser extent in these same tissues in females. We could speculate that *bab* is important for neuronal development in both males and females. However, its role in adults is probably different between sexes based on its differential expression. *LIM homeobox*, *Bgi12353B*, and *ago* were all highly expressed in adult male antennae along with *bab*, while *Bap18* was almost exclusively expressed in adult brain (Fig. 1a). *Bgi12353A* was expressed at low levels across all tissues and time points (not shown). A new set of quantitative PCRs, including 14 tissues from each strain, was conducted to test for strain-specific expression differences in tissues involved in pheromone signal processing, such as developing pupal antennae, adult antennae, and the adult brain. Expression levels were not statistically significantly different between E and Z strains, except for *ago* with significantly higher expression in adult E antennae for both sexes (Supplementary Fig. 3). *bab* had higher expression in Z male pupal antennae compared to E male pupal antennae and higher expression in E male adult antennae compared to Z male adult antennae. These differences were not statistically significant but are suggestive of a possible developmental delay of *bab* expression in males of the E strain. Functional activities of *bab1* and *bab2* duplicated transcription factor homologs in *D. melanogaster* are highly sensitive to changes in dose^18^ and expression time^19^; therefore, subtle differences in *O. nubilalis bab* expression might affect olfactory circuit development.

Whole mount in situ hybridizations compared cellular localized expression of *bab* and other genes involved in the pheromone response circuit during olfactory system development. We analyzed pheromone receptors OnubOR4 (detecting E11-14:OAc)^20^ and OnubOR7 (likely detecting Z11-16:Ald, an antagonist)^21^, and the odorant receptor co-receptor Orco^22,23^, which is obligately co-expressed with each odorant receptor (OR) in the same antennal OSNs for proper ligand gated channel function. Pupal antenna *bab* and *Orco* were co-expressed in the same OSN precursor cells, while in the adult, cells expressing *bab* were separate from OSNs expressing *OR7*, *OR4*, or *Orco* but directly adjacent (co-localized) in the same trichoid sensilla (Fig. 1b; Supplementary Fig. 4). These results indicate that neuronal cells are not yet fully differentiated in 4-day-old pupae, and that differential expression of *bab* may affect neurogenesis at this developmental stage to establish adult OSN functional topology.

### Further dissection of the *bab* gene

Two recombinant inbred lines had crossovers ensuring that the entire *bab* gene originated from Z (L165) or E strain (L205). Long-read genome sequences from these lines showed that each *bab* allele had five exons and a very large first intron (~293 kb), similar to other Lepidoptera. *bab* coding sequence contained three conserved domains: the BTB domain (bric à brac-tramtrack-broad complex domain) involved in dimerization, and the pipsqueak domain and the AT-hook that are both involved in DNA-binding^18,24^. Exons were verified from RNA-seq data, which also predicted transcripts containing a 78 bp insertion between exon 1 and 2 corresponding to an in-frame insertion of 26 amino acids compared to other lepidopteran species. We named this “exon 1.5” and its flanking introns “1A” and “1B.” Exon 1.5 was flanked by appropriate splice sites in both L165 and L205, but it could not be found in any genome or transcript sequence of any other examined insect. We were not able to reliably amplify exon 1.5 from cDNA suggesting it may be an intermediate splice variant removed during RNA maturation.

**Fig. 2:**
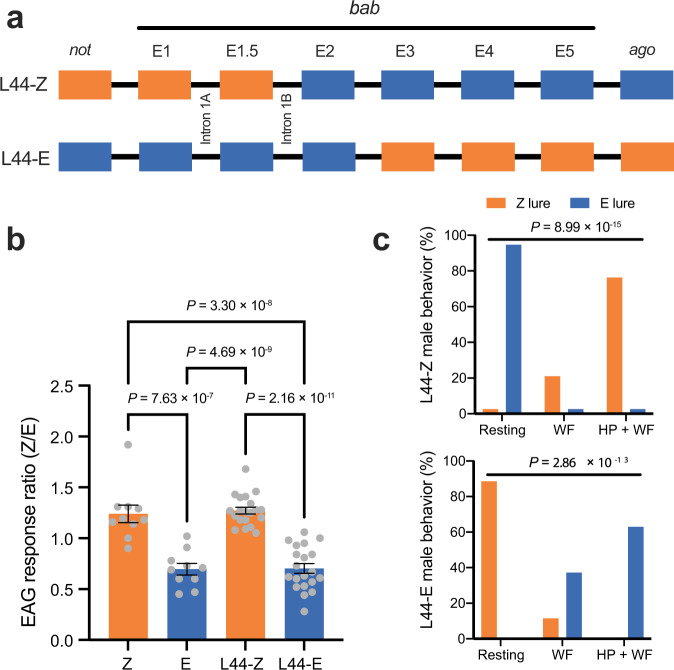
Electrophysiological and behavioral analysis of *bab*-recombinant lines. **a** Crossover points relative to exons of *bab* within lines L44-Z and L44-E. Boxes represent exons 1–5, orange gene regions originated from the Z-strain and blue from the E-strain. Locations of introns 1A and 1B are noted. Flanking genes *ago* and *not* are each represented by a single box. **b** Electroantennogram (EAG) response ratio of pure strain and *bab*-recombinant males. Data are presented as mean ± SEM of EAG response (in mV) to Z11-14:OAc divided by response to E11-14:OAc. Sample sizes of measured animals are Z-strain *n* = 10, E-strain *n* = 10, L44-Z *n* = 20, L44-E *n* = 20. Z-strain and Z-like responses are shown in orange, E-strain and E-like responses are shown in blue. *P* values report results of two-sided Tukey’s HSD post-hoc tests after an ANOVA (*F* = 38.67, df = 3, *P* = 1.13 × 10^−13^). **c** Wind tunnel responses of L44-E and L44-Z males to the Z-strain pheromone lure (97% Z-isomer, 3% E-isomer) are shown in orange and to the E-strain lure (1% Z-isomer, 99% E-isomer) in blue. *P* values report results of chi-square tests for L44-Z (*χ*^2^ (2, *n* = 38 animals) = 64.89) and L44-E (*χ*^2^ (2, *n* = 35 animals) = 57.76). Resting, no response to pheromone; WF wing-fanning response to pheromone, HP + WF wing-fanning and hair-pencil extrusion response to pheromone. Source data are provided as a Source Data file.

To test whether *bab* exclusively coincides with *Resp*, male-informative backcrosses were generated by crossing lines L165 and L205 to obtain new lines L44-E and L44-Z with crossovers between *not* and *ago*, and within *bab* (Fig. 2a). Phenotypes from these new lines localized *Resp* upstream of exon 2 in *bab*. Males of L44-E with *bab* exon 1 to exon 2 from the E-strain had an E-strain electroantennogram response (Fig. 2b), behavioral response in the wind tunnel (Fig. 2c) and single-sensillum response (Supplementary Fig. 5). Line L44-Z males, where exon 1–exon 1.5 were inherited from the Z-strain, had corresponding Z-strain responses (Fig. 2b, c, Supplementary Fig. 5).

To search for *bab* coding region polymorphisms linked to male response, sequences of the 669-bp exon 1 of *bab* were compared among 16 previously phenotyped lines, namely all 10 *Resp*-recombinant and *bab*-recombinant lines, four laboratory E or Z strains from Europe or United States, as well as one Z-strain and one E-strain US field population (Supplementary Methods). Neither of the two detected amino-acid substitutions were strain-specific differences that could underlie preference (Supplementary Data 1). Potential phenotypic effects of exon 1.5 were evaluated by using CRISPR/Cas9 to create a *bab* gene lacking a complete exon 1.5. Four homozygous mutant lines originating from E-strain-injected embryos had exon 1.5 deletions (Supplementary Fig. 6a), but all four lines had behavioral and electrophysiological responses similar to wild-type E strain males (Supplementary Fig. 6b, c).

### Genomic associations of preference in nature

**Fig. 3:**
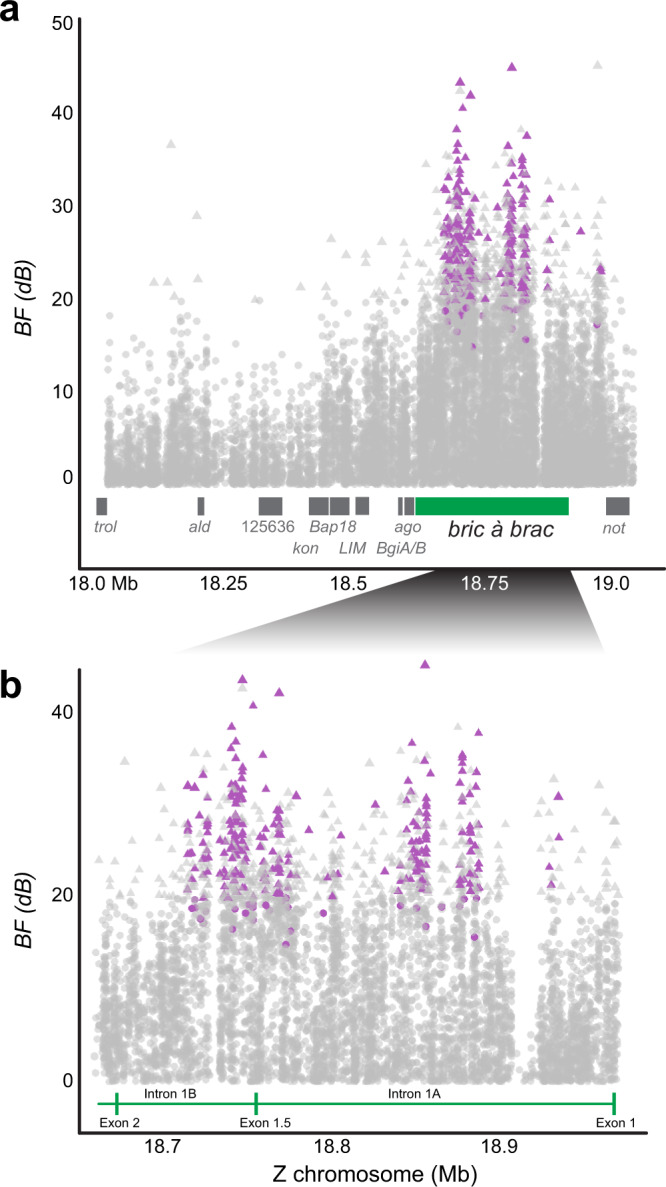
Polymorphisms associated with pheromone response in field populations. Bayes factor (BF) for polymorphism (SNP, indel, SV) association with pheromone trap after accounting for population structure plotted along the Z chromosome (in Mb). **a** Male *Resp* QTL region with candidate genes (gray) and *bab* (green). **b** The first intron of *bab*. (**a**, **b**) Bayes factor (BF) > 20 dB (triangle) and *eBP*_is_ > 2 (purple) indicate the strongest evidence for an association. Vertical green lines indicate *bab* exon boundaries, including rare splice variant exon 1.5. Note orientation is opposite of Fig. 2.

Parallel to QTL mapping, we conducted a GWAS of field collections of sympatric E and Z strain populations to identify specific mutations linked to preference variation. Male preference in the wild was measured as behavioral attraction to either Z or E pheromones released from paired pheromone traps 30 m apart. After controlling for population structure between two sympatric locations (Landisville, PA, USA: *n* = 16 males per trap; Rockspring, PA, USA: *n* = 15 males per trap; *n* = 62 males total), *bab* was the only gene on the Z chromosome strongly associated with pheromone blend attraction (Bayes factor > 20 dB, Bayesian *P*-value (*eBP*_*is*_) > 2; Fig. 3a; Supplementary Data 2). None of the associated polymorphisms (SNP, indels, SV) were within *bab* coding sequence, whereas 98.7% were located in intron 1A (*n* = 133 polymorphisms) or intron 1B (*n* = 96 polymorphisms) (Fig. 3b; Supplementary Data 2). Three polymorphisms were upstream of *bab* between *bab* and *not*. Preference had its strongest association with a 3-bp indel in intron 1A (BF = 45.26 dB, *eBP*_*is*_ = 3.27, *β* = 0.15). In expanded GWAS analyses that included more individuals and a third sympatric site (Bellona, NY, USA), we compared pool-seq genomic data of males from E pheromone traps homozygous for the E allele at *pgFAR* (*pgFAR-e/pgFAR-e* genotypes) (*n* = 31, 34, 41 males per site, 106 males total) to males in Z pheromone traps homozygous for the Z allele at *pgFAR* (*pgFAR-z/pgFAR-z* genotypes) (*n* = 25, 33, 39 males per site, 97 males total). *bab* showed the highest levels of differentiation across the genome (mean *F*_ST_ = 0.96; non-overlapping 1-kb windows; Supplementary Fig. 7a) and had the strongest association with preference according to Cochran–Mantel–Haenzel (CMH) tests of biallelic variants (CMH −log *P* > 64.84, FDR *q* < 7.73 × 10^−60^; Supplementary Fig. 7b).

### Genome scan of positive assortative mating

Asymmetric male preference for female pheromones in *O. nubilalis* is thought to contribute to a pattern of positive assortative mating^25,26^, which theory predicts will lead to two types of non-random genetic correlations at alleles controlling signals (here, the female pheromone blend) and preferences (here, the male preference for the female pheromone)^27^. First, alleles for signals will associate non-randomly with alleles for preferences, and second, a deficit of heterozygotes will occur at loci determining assortment. We conducted a genome scan to test these two predictions.

Since females homozygous for E *pgFAR* alleles produce E-biased pheromones, they will tend to attract E preferring males, whereas females homozygous for Z *pgFAR* alleles produce Z-biased blends and will attract Z preferring males^25^. If these divergent male preferences are sufficiently strong and lead to divergent patterns of mating, a positive genetic correlation can build up between signal and preference loci that might aid in the identification of genes underlying assortment. We evaluated this first key prediction by measuring linkage disequilibrium between the autosomal gene *pgFAR* and the *O. nubilalis* Z chromosome.

**Fig. 4:**
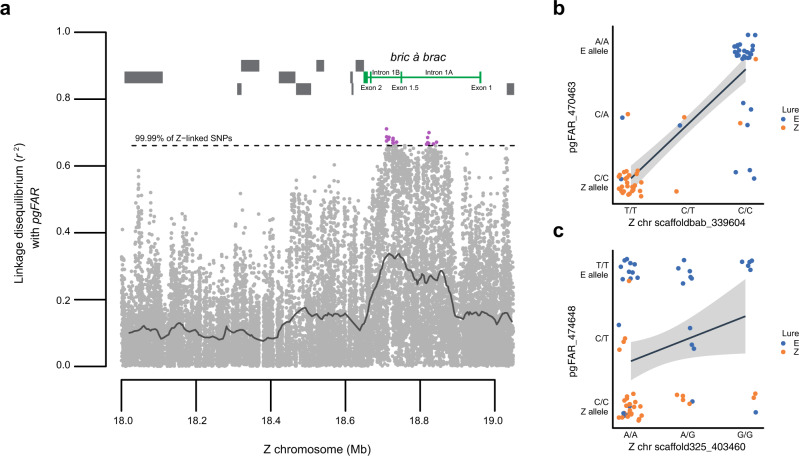
Linkage disequilibrium between the autosomal gene controlling female pheromone blend (*pgFAR*) and the Z chromosome. **a** Of amino-acid changing mutations detected at *pgFAR*, the one showing maximum *r*^2^ with each Z-linked polymorphism (281,385 SNPs) is plotted for the 18–19 Mb associated interval on the Z chromosome (22,689 SNPs) (*n* = 62 males). Purple points depict *r*^2^ values falling above the 99.99th percentile (dashed line; *r*^2^ = 0.66, 26 SNPs) for the ~21-Mb Z chromosome. *bab* gene structure (green) and neighboring genes (gray) are shown. The solid line depicts a sliding window average of *r*^2^ (1 kb with 100-bp step). **b** A *pgFAR* SNP underlying a leucine (Z allele) to isoleucine (E allele) substitution shows a maximum chromosome-wide *r*^2^ of 0.71 with a SNP within intron 1B of *bab* (position 18.71 Mb). All males homozygous for the E allele (A/A) at *pgFAR* were homozygous for the C SNP at *bab* (23 individuals). Most males homozygous for the Z allele at *pgFAR* (C/C) were homozygous for the T SNP at *bab* (27 of 31 individuals, 87%). *pgFAR* heterozygotes were more evenly associated with *bab* genotypes. 71% of males caught in the E trap were *pgFAR*^*A*^ *bab1*^*C*^ multilocus homozygotes (22 of 31) and 84% of males caught in the Z trap were *pgFAR*^*C*^ *bab1*^*T*^ multilocus homozygotes (26 of 31). **c** A representative *pgFAR* SNP and Z-linked SNP on scaffold 325 having an *r*^2^ value close to the Z chromosome median value of 0.06. Data (**b**, **c**) are presented as an estimated linear regression line (black) with a 95% confidence interval (gray band), and point color indicates the pheromone blend to which each field-caught male was attracted. Males caught using Z and E pheromone lures are shown in orange and blue, respectively. Source data are provided as a Source Data file.

One or more of 33 nonsynonymous substitutions at *pgFAR* is responsible for differential reduction of pheromone precursors into strain-specific Z or E blends^7,28^. Linkage disequilibrium was estimated as the squared correlation coefficient (*r*^2^) between these nonsynonymous SNPs and those located on the Z chromosome (*n* = 62 males total). *r*^2^ values between *pgFAR* alleles and 26 Z-linked SNPs fell above the 99.99th percentile (*r*^2^ > 0.66) of the Z chromosome and all occurred in either intron 1A or intron 1B of *bab* (Fig. 4; Supplementary Fig. 8). The maximum chromosome-wide *r*^2^ of 0.71 was attained between a SNP within *pgFAR* exon 5 and a SNP within *bab* intron 1B. Linkage disequilibrium between signal and preference genes caused by assortative mating are comparable to those caused by tight physical linkage, as roughly half (46%) of SNPs separated by 1 base pair of physical distance on the Z chromosome had estimated *r*^2^ values as high as those observed between physically unlinked *pgFAR* and *bab* loci (Supplementary Fig. 9).

The second prediction of a heterozygote deficiency yielded a similar result. *bab* contained the most significant deficit of heterozygous genotypes within any Z-linked gene (intron 1A: *P* = 2.47 × 10^−18^; *q* = 1.73 × 10^−12^), possessing the top 31 deviant polymorphisms (intron 1A = 17, 1B = 14) and nearly twice the number of significant polymorphisms of any other gene in the genome (5842, FDR *q* < 0.01) (Supplementary Fig. 10).

## Discussion

Two independent approaches, namely fine-scale QTL mapping of electrophysiology and behavior with controlled crosses in the laboratory, and population genetic analysis of behavior and assortative mating with genome-wide data from nature, have converged on non-coding sequence of *bric à brac* as responsible for variation in male preference between the two pheromone strains of the European corn borer. Nothing is known of the olfactory function of *bric à brac* in the European corn borer or any other lepidopteran. Before proposing a hypothesis for the mechanistic role of this transcription factor we need to consider physiological components of pheromone communication.

In antennae, OR genes responsible for pheromone detection were attractive candidates, because pheromone components must bind to these membrane proteins in OSNs in the antennal sensillum to be perceived by the insect. Specific amino acid substitutions in ORs of different *Ostrinia* species^20^ can dramatically shift binding specificity, and such modifications may underlie other species differences in pheromone sensing and response, as they do between noctuid *Heliothis* moths^13^. However, there is little evidence that sex-linked OR genes expressed in the antennae differ significantly in Z and E *Ostrinia* strains. A detailed study of expression using in situ hybridization found a single type of sensillum containing three OSNs, with expression of sex-linked ORs essentially the same in both strains^21^. The fact that the OR cluster can be recombinationally separated from Z or E male behavior proves that neither protein sequence differences nor *cis*-acting regulation of ORs can explain changes in behavioral response^9,14,15^.

More relevant as a physiological locus of asymmetric preference are features of olfactory processing between the antenna and the antennal lobe of the brain. The three sensillar OSNs can be distinguished by their spike amplitudes, which correlate with the size of OSN dendrite projecting into the sensillar lymph^29,30^. In the Z-strain, the largest spiking neuron expresses Z11-14:OAc-detecting OR6 and is sensitive to Z11-14:OAc, the smallest spiking neuron expresses E11-14:OAc-detecting OR4 and is sensitive to E11-14:OAc, and the neuron with intermediate spike amplitude expresses three or four other ORs and responds to several behavioral antagonists including (Z)-9-tetradecenyl acetate (Z9-14:OAc)^21^. In the E-strain, the largest and smallest spiking neurons have reversed OR expression and pheromone specificity. In addition to these functional differences are neuroanatomical shifts involving the pheromone-sensitive macroglomerular complex in the antennal lobe. In the Z-strain, axons from the Z11-14:OAc-responding OSNs converge on the larger medial glomerulus, while axons from the E11-14:OAc-responding OSNs target the smaller lateral glomerulus. In the E-strain, this topology is reversed^31^.

Each instance of reversed neural architecture behaves as a sex-linked trait. Spike amplitude variation associates with a Z chromosomal region containing *bab* in our recombinant inbred lines, consistent with previous work indicating at least partial correlation with sex-linked behavioral response^32,33^, and in hybrids and backcrosses studied previously, neuroanatomical variation is sex linked, with connectivity exhibiting E-dominance but glomerulus volume showing additive effects^34^. These neuroanatomical studies did not measure male behavioral responses or inheritance of Z linked genes in backcrosses; we did not study anatomy in our backcrosses or recombinant inbred lines. But elimination of the OR cluster on genetic grounds makes the connection of the *bab* polymorphism to OSN specificity or re-wiring most likely to explain male preference.

In *D. melanogaster*, a tandem duplication has resulted in two genes at the *bab* locus, *bab1* and *bab2*, which play a variety of roles in development. *bab* loci and other transcription factors are part of a gene regulatory module that patterns the *Drosophila* antennal disc into concentric zones that specify olfactory neuron identity^19^. A well-studied sexually dimorphic pattern of adult abdominal pigmentation is also influenced by *bab1*, due to variation in regulatory elements in the conserved large first intron that alter *bab1* expression in pupae^35–37^. Therefore, we suggest that differences in *cis* regulatory elements in the first intron of *bab* alter the temporal or spatial pattern of expression of the gene in the developing *O. nubilalis* olfactory system, with downstream effects on receptor specification of OSNs in the antenna or axonal targeting to the antennal lobe of the brain.

New lepidopteran olfactory preferences are traditionally expected to arise primarily at the level of signal detection rather than signal processing, because high specificity and selectivity provided by ORs and their ligands can minimize broader impacts of genetic change on neural architecture and perception of the wider olfactory environment^2,38^. Accordingly, there is a plethora of studies on the function and evolution of ORs and olfactory preference, while fewer studies have focused on other factors. Our results point to a distinctly different target of olfactory preference evolution, which involves altering of sensory neuronal identity in the antennal sensillum through targeting by a transcription factor. That two very distinct mechanisms have been discovered in *Heliothis* and *O. nubilalis*, two of the limited number of moth species amenable to QTL mapping, indicates that there is not a single evolutionary trajectory. Apparently, evolution can be accomplished by changes of direct sensing of pheromones as in *Heliothis*^13^, or in a manner reminiscent of other sensory modalities (e.g., visual, auditory^39,40^), in *O. nubilalis* by altered signal processing, despite potential pleiotropic effects that may be associated with transcription factor mutations, including to other parts of the olfactory circuit^19,38,41^. Mechanisms of lepidopteran olfactory evolution therefore appear more diverse than commonly believed, with fewer constraints and more properties in common with other sensory modalities. Our results provide new possibilities to explore how *bab* differences precipitate altered sensory circuit architecture to allow male preferences to diverge, while remaining flexible to possible influences on perception of odors in the broader environment (e.g., host plant, male sex pheromones). *bab* has not, to our knowledge, been previously associated with pheromone communication, but this well-conserved transcription factor could potentially contribute to sexual response divergence in other Lepidoptera.

Discriminating sexual activity is fundamental to animal behavior, population divergence, and speciation^2,3,5^, but natural variation in mate choice has been challenging to genetically dissect because it involves identifying the loci responsible for an interaction between one animal’s sensory and neural system, and another animal’s complex of signals and displays. Although mate choice is mediated by various sensory modalities, emphasis has been placed on changes in behavioral response to new pheromones because chemical communication is generally regarded as both ancient and widespread^3^. Our study implicates the sex-linked transcription factor *bab* as a modifier of neural architecture and the fates of OSNs underlying pheromone detection in antennal sensilla, leading to saltational shifts in sex-pheromone preference in males. *bab* along with the previously described autosomal gene responsible for shifts in female pheromone structure, *pgFAR*^7^, account for the capacity of diverged moth populations to discriminate among potential mating partners^25^.

Identification of both *bab* and *pgFAR* genes is an important starting point for studying the microevolutionary dynamics of mate choice. Divergence of mate choice may often require signals and preferences to genetically covary and be evolutionary linked^3,5^, particularly if they are under independent genetic control and not physically linked^9,13^. Indirect estimates of genetic covariance using signal-preference phenotypic correlations can be weak or absent^2,5^. However, *bab* and *pgFAR* allow us to precisely estimate covariance directly at the genetic level by measuring the correlation between signal and preference genes. Sex-linked *bab* intronic alleles co-occurred with autosomal E and Z *pgFAR* pheromone alleles ~80% of the time, suggesting that diverged preferences observed in the laboratory^25^ are strong enough in nature to facilitate assortative mating and mate choice divergence, despite the separation of signal and preference loci on different chromosomes^9^. Although pleiotropy and physical linkage of signals and preferences^39,42^, or a simple matching mechanism^43^, might increase the likelihood of assortative mating evolution^3,5^, the absence of these features has apparently not been an obstacle to coevolutionary divergence of female pheromone production and male behavioral response genes. Future application of a combination of experimental manipulation, evolutionary inference, and population genetic modeling to *bab* and *pgFAR* will allow new opportunities to address the many theories proposed to explain the remarkable diversity of the moth sex pheromone system.

## Methods

### Generation of *Resp*-and *bab*-recombinant lines

Male informative backcross (BC) families using *O. nubilalis* Slovenia and Hungary strains^15^ were generated that exhibited fixed recombination between the flanking genes of the *Resp* region, *trol* and *not*. ZE and EZ hybrid males were backcrossed to a Z-strain female to generate backcross 1 (BC1) (Supplementary Fig. 1a, b). Recombinants between *trol* and *not* were identified via polymerase chain reaction (PCR) (Supplementary Methods, Supplementary Table 1), and crossed to Z-strain individuals to obtain BC2 (Supplementary Fig. 1c). BC2 individuals were genotyped to detect recombinants, then mated with each other to generate inbred 1 (IB1) crosses (Supplementary Fig. 1d). IB1 adults with the desired genotype were mass reared to obtain IB2 (Supplementary Fig. 1e). IB2 families that originated from a BC1 male cross were fixed homozygote recombinants, whereas BC1 female cross descendants were genotyped and inbred again to obtain fixed recombinant homozygotes (Supplementary Fig. 1f, g). Nine *Resp*-recombinant lines had one recombination point between homozygous *trol* and *not* genes (L165, L173, L185, L190, L195, L205, L215, L220, L237). *bab*-recombinant lines exhibited fixed recombination between *bab*’s flanking genes, *ago* and *not*, and were generated using the two homozygote recombinant lines L165 with Z-strain phenotype and L205 with E-strain phenotype. Single pair matings between L165 females and L205 males were set up to obtain hybrid males, which were backcrossed to L165 females. The BC individuals were screened with PCR (Supplementary Methods) to select recombinant adults that were used for inbred mass rearing. The PCR selection process continued until two fixed homozygote populations were established, i.e. line L44-Z and line L44-E (Fig. 2a).

### Genomic sequencing of *Resp*-recombinant lines

A pool of 10 male pupae of lines L165 and L205 were homogenized in liquid nitrogen using mortar and pestle and DNA extractions were performed with QIAGEN Genomic-tip 100/G and the Genomic DNA Buffer Set (Qiagen, Hilden, Germany) according to the manufacturers’ instructions, but extending incubation times with buffer G2 containing proteinase K and RNase A to 12 h. HMW genomic DNA was sent to GATC Biotech for sequencing. Sequencing was done using an Illumina HiSeq2500 instrument, obtaining ~200 Mio paired end (2 × 150 bp) sequences per *Resp*-recombinant line. Shotgun genome assemblies were generated using the CLC Genomics Workbench v10.1. For PacBio sequencing, HMW genomic DNA was isolated from individual pupae of lines L165 and L205 by the Max Planck-Genome Centre Cologne (MPGCC) using the Qiagen MagAttract HMW DNA Kit. Sequencing of the size-selected HMW genomic DNA of each strain further purified with AMPure beads was performed at the MPGCC on a PacBio Sequel instrument. PacBio reads for both recombinant lines were assembled separately using the HGAP4 assembly pipeline implemented in the SMRT analysis software with standard settings. After genome sequencing of lines L165 and L205, primers were designed which amplified line-specific size polymorphisms and used to narrow down the breakpoint within all *Resp*-recombinant lines (Supplementary Methods, Supplementary Table 1).

### Phenotyping with wind tunnel assays

Wind tunnel experiments were conducted with 0–5-day-old unmated males in a 2.5 × 1 × 1 m wind tunnel at 20–25 °C, 70% humidity, 30 cm/s airflow, and 26% red light. Synthetic lures (Z-strain lure: 97% Z11-14:OAc + 3% E11-14:OAc; E-strain lure: 99% E11-14:OAc + 1% Z11-14:OAc) diluted Z11-14:OAc and E11-14:OAc (purity of ≥99%, Pherobank, Wijk bij Duurstede, Netherlands) with hexane to 30 µg per lure. Blend quality and quantity was confirmed with gas chromatography. Pheromones were applied to rubber septa (Thomas Scientific, Swedesboro, NJ, USA) and stored at −20 °C. Individual males were placed in a small cylinder (10 cm, 3.2 cm diameter) covered with netted cloth at both ends permitting flow of odorized air. After placing the cylinder at the downwind end of the wind tunnel, male behavior, i.e. (1) resting (=no response), (2) wing fanning (=medium response), and (3) hair-pencil extrusion (=highest response), was recorded using setup adapted from Koutroumpa et al. ^15^, Supplementary Fig. 11). Each male was exposed to one blend for 60 s, kept for 30–60 min in the tunnel without any odor, and then the opposite blend was tested. Blends testing order was switched between experimental days. Statistical analysis was performed with R version 3.6.1^44^ using Fisher’s Exact or Chi-squared test. To complement behavioral phenotypes, electrophysiological phenotypes (electroantennogram (EAG) and single sensillum recordings (SSR)) of *bab*-recombinant and CRISPR lines (described below) were recorded (Supplementary Methods).

### RNA isoform identification

De novo transcriptomes of US laboratory populations^45^ were constructed using Trinity^46^ separately for E- and Z-strain individuals following methods in Levy et al. ^47^ to identify all splice variants of candidate genes. RNA was isolated from larval heads^45^, adult female heads^47^, or from whole pupae newly reported here. Briefly, RNA was extracted from samples using RNeasy kits (Qiagen, Hilden, Germany), then quantified with a Nanodrop (Thermo Scientific, Wilmington, DE, USA) and Qubit Broad Range RNA assays (Life Technologies, Carlsbad, CA, USA). cDNA libraries were prepared from mRNA using the TruSeq Sample Prep Kit v2 Set A (Illumina Inc., San Diego, CA, USA) using 1 mg total RNA, and prepared libraries were quantified using the Qubit High Sensitivity DNA assay. Libraries were quantified a second time on an Agilent Bioanalyzer (Santa Clara, CA, USA). Libraries were run on an Illumina HiSeq 2500, located at the Tufts University Core Facility for Genomics (Boston, MA, USA) to generate 100 bp single-end reads. Single-end reads were assessed for quality using the FastQC program (http://www.bioinformatics.babraham.ac.uk/projects/fastqc). Sequences were then trimmed using Trimmomatic version 0.35 to remove adapter sequences, bases with low sequence quality, and any reads that were shorter than 36 base pairs. FastQC reports were generated for each file again to confirm post-trimming quality. Mitochondrial DNA and ribosomal RNA sequences were removed using Bowtie2^48^ by aligning against known mtDNA sequences and identical reads were collapsed prior to assembly (but counts retained) using the FastX Toolkit version 0.013 (http://hannonlab.cshl.edu/fastx_toolkit). The transcriptome was assembled de novo using Trinity^46^ and a k-mer length of 25. The longest transcript for each component were retained using custom scripts.

### Reverse-transcription quantitative PCR (RT-qPCR)

Six genes in the *Resp* region between *kon* and *not* (*Bap18, LIM, Bgi-A, Bgi-B, ago*, *bab*), plus *Orco* and *OnubOR6* were analyzed for their expression ratio in different tissues of E-strain and Z-strain individuals of European laboratory populations. Stages and tissues include: 5th instar larvae (antennae, head without antennae, thorax, abdomen), prepupal instar (head, thorax, abdomen), 2- and 4-day-old male and female pupae (antennae), 2-day-old male and female adults (antennae, brain, 1st pair of legs, 2nd plus 3rd pair of legs, abdomen). Expression ratios of *bab* were additionally evaluated for 7-day-old male and female pupal antennae as well as for 7-day-old male and female antennae and brains. Due to the large number of samples needing to be tested for expression simultaneously, a first qPCR was run comparing all tissues within each strain (Fig. 1a). At a next step only most expressed and most related tissues to the scientific question (i.e., antennae and brain) were included and comparisons were made simultaneously for the two strains (Supplementary Fig. 3). Three biological replicates of each of 27 sample types were collected during the second hour of scotophase from each strain. Total RNAs were extracted from each tissue using a Trizol/Chloroform approach followed by RNeasy Micro Kit purification (QIAGEN). Single-stranded cDNA synthesis was performed from 1 μg total RNA with iScript Reverse Transcription Supermix for RT-qPCR from BioRad (Hercules, CA, USA). Three control genes, (*GAPDH*, 18S rRNA, *rpL8*) were tested for stability between samples, and *rpL8*^49^ was chosen for final comparisons. Gene-specific primers designed using “Primer 3”^50^ amplified 100–200 bp fragments (Supplementary Table 2). qPCR reactions were performed using Sso Advanced Universal SYBR Green Supermix (BioRad) in a total volume of 12 μl with 3 μl cDNA (or water as negative control or RNA for controlling the absence of genomic DNA) and 0.25 mM of each primer. cDNA amplifications were performed in a BioRad CFX96 Real-Time System using a gradient of annealing temperatures for each gene of interest. Three gradient temperatures were tested per gene on a 4-fold dilution series, 1/4–1/128 of a sample representative cDNA pool [*E* = 10 (−1/slope)] for relative quantification of the same gene in all other cDNA samples. Two replicates of each dilution were tested. A melting curve ramp (65–95 °C: Increment 0.5 °C/5 s) was generated to confirm that reactions did not produce nonspecific amplification. The final protocol included a denaturation step at 95 °C for 3 min followed by 40 cycles of amplification and quantification (denaturation at 95 °C for 10 s and annealing for 30 s at temperatures given in Supplementary Table 2 for each primer pair). Reactions were performed in two technical replicates. After confirming similar amplification efficiencies of target and control gene, expression levels were calculated relative to *rpL8* expression and expressed as the ratio = *E*^(−Cq *Resp candidate*)^/*E*^(−Cq rpL8)51^. Statistical comparisons between strains, sexes, and tissues for each gene were assessed using one-way analysis of variance (ANOVA), followed by honest-significant difference (HSD) tests (post hoc Tukey’s test). A Benjamini–Hochberg multiple-test correction was applied over the genes tested.

### Targeted mutagenesis of *bab* exon 1.5

Nine RNA guides were designed against intron 1A, exon 1.5, and intron 1B of *bab* (Supplementary Table 3) using the CRISPOR gRNA design tool cripsor.tefor.net and the *O. nubilalis bab* genomic DNA sequence as target. Guide sequences were subcloned in DR274 (http://www.addgene.org/42250) derived vector. Plasmids were digested by DraI, purified, and transcribed using HiScribe T7 high yield RNA synthesis kit (New England Biolabs). Reactions were purified using EZNA microelute RNA clean-up kit (OMEGA Biotek). *Streptococcus pyogenes* Cas9 protein, bearing three nuclear localization sequences, was provided by TacGene (Paris-France)^52^. Nine different guide RNAs were designed; three targeting exon 1.5, three in the preceding intron, and three in the following intron. Aliquots of sgRNA were denatured at 80 °C for 2 min and then left on ice for 2 min before mixing them with the equivalent amount of Cas9 for a sgRNA:Cas9 complex ratio of 1.5:1. Concentrations of the sgRNA are given in Supplementary Table 3 and the Cas9 was 30 µM (Sp-Cas9-NLS-GFP-NLS). The complex was formed at room temperature (RT) for 10 min. sgRNA:Cas9 complexes were formed separately for each sgRNA to ensure that Cas9 would bind equally to each sgRNA. These were combined as desired and placed on ice. Eggs of either strain from the European populations were injected (using an Eppendorf FemtoJet 4i injector) within 0.5 h after oviposition to target the one cell embryo stage. We injected three combinations of sgRNA (Supplementary Table 3) in order to create a deletion 5′ of exon 1.5 (KO1), a deletion 3′ of exon 1.5 (KO2), or a complete deletion of exon 1.5 (DEL). Injected eggs were reared to adulthood and genotyped. DNA of adult legs was extracted^51^ and amplified with Terra™ PCR Direct Polymerase Mix (Takara Bio Europe) using primer Bab-Z/E-i01-F9 (GTGCATTTCCTGCTTATGA) on intron 1, Bab-E-i01-R10 (AATTTGCCCCTAAGTGTACC) on intron 1.5, and the following program: 98 °C for 2 min, 35×(10 s at 98 °C, 15 s at 60 °C, 30 s at 68 °C). Size polymorphism were detected with agarose gel analysis and confirmed by Sanger sequencing (Macrogen, Amsterdam). Sequences were aligned using SEQUENCHER™ 4.7 (Gene Codes Corporation, Inc.). Heterozygote G0 adults with mutations were crossed to adults from the wild type rearing. G1 heterozygote males and females carrying the same mutation were crossed to obtain homozygote G2 mutants. Four G2 CRISPR lines were established: lines L46 (KO1), L72α (KO2), L72β (KO2), and L73 (KO2). Males of all CRISPR lines were phenotyped using EAG (Supplementary Methods) and wind tunnel assays.

### Whole mount in situ hybridization

Male *O. nubilalis* whole antennae were mounted and in situ hybridized with two RNA probes simultaneously. *bab* digoxigenin-labeled antisense riboprobe, was generated using a Sp6/T7 RNA transcription system (Roche) and linearized recombinant pCRII-TOPO plasmids (TOPO TA cloning kit Invitrogen) following manufacturer’s protocols. *Orco, OR4*, *OR6*, and *OR7* probes are the same preparations that were used in ref. ^21^. Two color double in situ hybridization with two different antisense RNA probes (digoxigenin-labeled or biotin-labeled probes), as well as visualization of hybridization were performed as reported previously^21,53^ and described below. Antennae of 1–2-day-old Z-strain and E-strain male moths from the European laboratory populations were dissected by first cutting off the tips. The remaining antennal stem was further cut into smaller pieces of 5–15 antennal segments. The same procedure was done for 4-day-old pupal antennae that were extracted underneath the pupal cuticle, which was broken and lifted at antennal base so that the antenna could be pulled out with forceps.

DIG-labeled probes were detected by an anti-DIG AP-conjugated antibody in combination with HNPP/Fast Red (Fluorescent detection Set; Roche); for biotin-labeled probes the TSA kit (Perkin Elmer, Boston, MA, USA), including an antibiotin–streptavidin–horseradish peroxidase conjugate and FITC tyramides as substrate was used. All incubations and washes were made in a volume of 0.3 mL (unless otherwise stated) in 0.5 mL tubes with slow rotation on a small table rotor at RT or in a hybridization oven (Bambino, Dutcher) when heating was needed. Antennal fragments were fixed in 4% paraformaldehyde in 0.1 M NaCO_3_, pH 9.5 for 24 h at 4 °C (PF1) followed by washes at RT for 1 min in phosphate-buffered saline (PBS: 0.85% NaCl, 1.4 mM KH_2_PO_4_, 8 mM Na_2_HPO_4_, pH 7.1), 10 min in 0.2 M HCl and 2 min in PBS with 1% Triton X-100. Antennal fragments were then incubated for 3 h in whole mount hybridization solution (50% formamide, 1% Tween 20, 0.1% CHAPS, 50 µg/mL yeast tRNA, 5× SSC, 1× Denhart’s reagent and 5 mM EDTA, pH 8.0) at 55 °C. Hybridization, using one DIG-labeled and one biotin-labeled probe, took place at 55 °C. Prior to hybridization, probes were diluted to adequate ratios (final volume 200 µL) in hybridization buffer (50% formamide, 10% dextran sulfate, 2× SSC, 0.2 µg/µL yeast tRNA, 0.2 µg/µL herring sperm DNA) and heated for 10 min at 65 °C. After heating, the probes were kept on ice for at least 5 min before use. Post-hybridization antennal fragments were washed four times for 15 min in 200 µL of 0.1× SSC (1× SSC = 0.15 M NaCl, 0.015 M Na-citrate, pH 7.0) at 60 °C then treated for 16 h in 5 mL of blocking solution (10 g blocking reagent from Roche in up to 100 mL maleic acid solution: 0.1 mol/L maleic acid and 0.15 mol/L NaCl) in 45 mL TBS and 150 µL Triton X-100 at 4 °C. The next step was to incubate fragments for 48 h with an anti-dioxigenin alkaline phosphatase-conjugated antibody (Roche) diluted 1:500 and with a streptavidine horse radish peroxidase-conjugate diluted 5:500 in blocking solution in TBS prepared as previously. After washing five times for 10 min in TBS, 0.05% Tween, antennal fragments were rinsed in DAP-buffer (100 mM Tris, pH 9.5, 100 mM NaCl, 50 mM MgCl_2_), after which hybridization signals were visualized using HNPP (Roche; 1:100 in DAP-buffer, pH 8.0) incubations for 15 h at 4 °C. After washing five times for 10 min in TBS, 0.05% Tween, antennal fragments were incubated for 18 h with the TSA kit substrates (Perkin Elmer, MA, USA): 2% Tyramide in amplification diluent. After a last set of washes, five times for 10 min in TBS, 0.05% Tween, antennal fragments were mounted in 1/3 PBS/glycerol and specific antennal cell stainings were observed with a Zeiss (Oberkochen, Germany) LSM 700 confocal laser scanning microscope (MIMA2 Platform, INRA, France, 10.15454/1.5572348210007727E12). Images were arranged in Powerpoint (Microsoft) and Adobe Illustrator (Adobesystems, San Jose, CA, USA) and were not altered except adjusting brightness or contrast for uniform tone within a figure.

### Phenotyping pheromone preference in nature

Pheromone trapping in North America was used to collect wild E-pheromone and Z-pheromone preferring males using Scentry *Heliothis* traps baited with synthetic E (“New York”) and Z (“Iowa”) lures (Scentry Biologicals, Billings, MO, USA). Traps were placed directly next to sweet corn fields and males were collected from each trap every 1–2 weeks and stored at −20 °C. Lures were replaced every 2 weeks. Trapping of >20 males from each E and Z trap was done at three sympatric sites between 2010 and 2012 (Supplementary Table 4). Tissues were moved from −20 °C within 3 months of collection to at −80 °C for long-term storage. DNA was isolated from both Pennsylvania sites by grinding frozen tissues and using the Qiagen DNeasy tissue protocol (Qiagen, Germantown, MD, USA) without vortexing preserve high molecular weight DNA. DNA isolation of samples from Bellona, NY was conducted with Qiagen genomic tips (20 G). All samples were treated with Qiagen RNase. DNA concentrations were quantified using Qubit prior to sequencing.

### Individual genome resequencing of field moths

Individual resequencing data were collected for 31 E-trapped and 31 Z-trapped individuals from two sites (Rockspring, PA, USA (*n* = 15 per trap), and Landisville, PA, USA (*n* = 16 per trap); Supplementary Table 5). Landisville, PA, Z-trap data were originally described by Kozak et al. ^54^; all other data are new. Libraries were prepared using Illumina TruSeq (Illumina Inc.) and sequenced on an Illumina NextSeq using 150 bp paired-end sequencing at Cornell University. Trimmed genomic data were analyzed using the GATK best practices pipeline^55–57^ with data aligned to the repeat-masked genome reference (GenBank BioProject: PRJNA534504; Accession SWFO00000000^54^) using bwa^58^, sorted and filtered using Picard and samtools to remove duplicates and reads with a mapping quality score below 20. SNPs and small indels were called using GATK Haplotype caller (joint genotyping mode) after realigning around indels and filtered using recommended GATK filters^57^. Large structural variants (SV) were called from aligned bam files using information from split paired end reads using split reads and anomalies in pair orientation and insert size in Delly2^59^ (https://github.com/dellytools/delly); these structural variants included indels (>300 bp), translocations, and inversions. Delly2 was run on all individual files, these were merged to a consensus SV file and genotypes were reassessed.

BayPASS 2.1^60^ was used to identify SNPs associated with pheromone trap while controlling for population demography in the individual resequencing data using allele frequencies for our four populations to test the association with pheromone trap (*Z* = 1, *E* = −1) using the STD model. As described in Kozak et al. ^54^, significantly associated polymorphisms had *XtX* above the 0.001% quantile of pseudo-observed data of simulated “neutral” loci, BF > 20 dB^61^, and *eBP*_*is*_ > 2 (equivalent to *P* value < 0.01 for *β* = 0)^60,62,63^. We expected the demographic history of the sex chromosome to be different from that of the autosomes, so we ran analyses with only Z chromosome loci. *F*_ST_ was calculated in vcftools v0.1.16^64^. Plots were created in R using packages qqman^65^ and ggplot2^66^.

### Pooled genome resequencing of field moths

Pooled samples were created by including equal amounts of DNA from all males caught within the same pheromone trap that were also homozygous *pgFAR* genotypes, as determined by a diagnostic *Taq*1α restriction digest^67^. At each of three sites (Rockspring, PA, Landisville, PA, Bellona, NY), separate E-preferring *pgFAR-e/pgFAR-e* (E-strain) and Z-preferring *pgFAR-z/pgFAR-z* (Z-strain) pooled libraries were prepared as above and uniquely indexed E and Z pools from each site were sequenced on a single Illumina sequencing lane (*n* = 25–41 males per trap per site; 203 males total). Bellona, NY, and Landisville, PA, pools were sequenced on an Illumina HiSeq3000 at the Iowa State University DNA Facility (Ames, IA) using 150 bp paired-end sequencing while Rockspring, PA, pools were sequenced using 100 bp single end sequencing. Rockspring, PA, and Landisville, PA, data were originally described in ref. ^68^, whereas Bellona, NY, data are new. Genomic reads were trimmed using Trimmomatic v.35 to remove Illumina adapters (TruSeq2 single-end or TruSeq3 paired-end), reads with quality <15 over a sliding window of 4 and reads <36 bp long. Reads were aligned using Bowtie2^48^ and genomic positions were determined as described above. Aligned reads were sorted and filtered using Picard and samtools to remove duplicates and reads with a mapping quality score below 20. Samtools was used to identify single nucleotide polymorphisms (SNPs) in populations^58^.

Scripts from the Popoolation2 package were used to filter SNPs (removing SNPs near small indels, and those with rare minor alleles that did not appear twice in each population), calculate allele frequency and *F*_ST_ between strains^69,70^. We ran the CMH test to identify consistent differences in allele frequency among independent sympatric E and Z pools from a given site^36^. CMH comparisons were done on individual SNPs that passed a Woolf heterogeneity test (read coverage minimum 10, maximum 200)^71^. *P* values were corrected using a genome-wide using false discovery rate (FDR) in the fdrtool package in R^72^. We also calculated the mean *F*_ST_ for each of the 3 pairs of E and Z strains by population over 1 kb windows.

### Genome scan of positive assortative mating

Using individual genome resequencing data, we conducted a scan for two signatures of assortative mating: (a) elevated linkage disequilibrium with the autosomal signal locus *pgFAR* and (b) a deficit of heterozygote genotypes. LD was calculated as the squared correlation coefficient (*r*^2^) between 33 missense mutations in *pgFAR*^28^ and the Z chromosome using vcftools after genotype phase was imputed with Beagle 5.0^73^ and data were filtered for SNPs with minor allele frequency >0.05. All but two nonsynonymous *pgFAR* SNPs are fixed between strains^7^ and should have identical correlations with Z loci; however, estimates of LD varied due to changes in genome sequencing coverage (e.g., 2 of 33 nonsynonymous mutations at *pgFAR* lacked coverage). To estimate how LD typically varies across unlinked regions, *r*^2^ values were calculated from ≥50 kb chromosome-assigned scaffolds for variants separated by at least 1 Mb or located on different chromosomes (*n* = 1350 SNPs). To estimate how LD varies by physical distance on the Z chromosome, *r*^2^ values were calculated from variants within 15 kb regions haphazardly sampled across all Z scaffolds ≥ 50 kb (*n* = 57). A deficit of heterozygous genotypes was tested using an exact test as implemented in vcftools “hwe” function with a genome-wide FDR correction.

### Reporting summary

Further information on research design is available in the Nature Research Reporting Summary linked to this article.

## Supplementary information

Supplementary Information

Reporting Summary

Description of Additional Supplementary Files

Supplementary Data 1

Supplementary Data 2

## Data Availability

Genome data are available from GenBank under multiple BioProjects. Pooled resequencing data can be found at BioSample, PRJNA361472 (Rockspring, PA, Landisville, PA) and BioSample, PRJNA655940 (Bellona, NY). Individual resequencing data can be found at BioSample, PRJNA540833 (Landisville, PA, Z trap) and BioSample, PRJNA656178 (Landisville, PA, E trap; Rockspring, PA, E trap; and Rockspring, PA, Z trap). New RNA-seq data are available at Bioproject, PRJNA704411. Confocal images are available at BioStudies under accession number S-BSST601. Source data are provided with this paper.

## Source data

Source Data
