# Supplementary material for: bric à brac controls sex pheromone choice by male European corn borer moths
Source: Nat Commun. 2021 May 14;12:2818. doi: 10.1038/s41467-021-23026-x (PMC8121916; doi:10.1038/s41467-021-23026-x)
Supplement: Supplementary file 1 — Supplementary Information [file 41467_2021_23026_MOESM1_ESM.pdf]

bric à brac controls sex pheromone choice by male European corn borer moths

Authors: Melanie Unbehend[1], Genevieve M. Kozak[2,7], Fotini Koutroumpa[3,4], Brad S. Coates[5], Teun Dekker[6], Astrid T. Groot[1,3], David G. Heckel[1*], Erik B. Dopman[2*]

Affiliations:

[1]Department of Entomology, Max Planck Institute for Chemical Ecology, 07745 Jena, Germany

[2]Department of Biology, Tufts University, 200 Boston Avenue, Ste. 4700, Medford, MA 02155, USA

[3]Institute for Biodiversity and Ecosystem Dynamics, University of Amsterdam, 1098 XH Amsterdam, the Netherlands

[4]INRAE, Sorbonne Université, CNRS, IRD, UPEC, Université Paris Diderot, Institute of Ecology and Environmental Sciences of Paris, Route de Saint-Cyr, 78026 Versailles Cedex, France

[5]USDA-ARS, Corn Insects and Crop Genetics Research Unit, 532 Science II, 2310 Pammel Drive, Ames, IA 50011, USA

[6]Department of Plant Protection Biology, Swedish University of Agricultural Sciences, SE-23053 Alnarp, Sweden

[7]Present Address: Department of Biology, University of Massachusetts Dartmouth, Dartmouth, Massachusetts 02747, USA

*Corresponding author.

These authors contributed equally: Melanie Unbehend, Genevieve M. Kozak, Fotini Koutroumpa

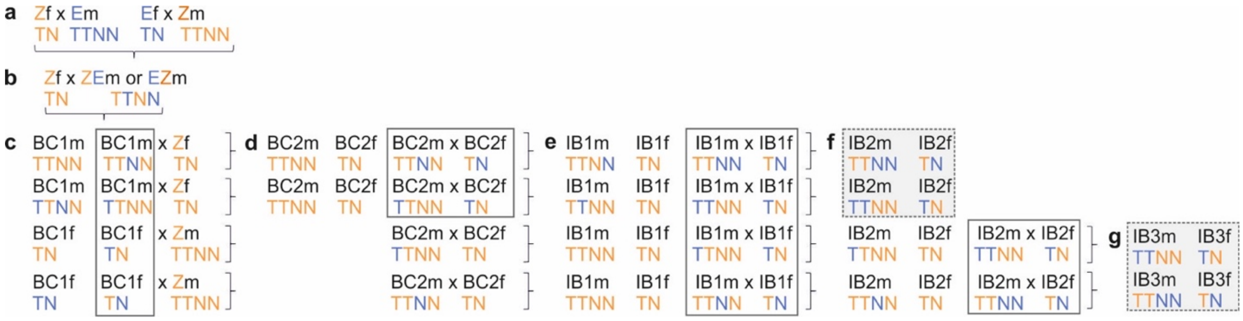

Supplementary Figure 1 | Crossing scheme to generate *Resp*-recombinant inbred lines with crossovers between the genes *trol* (T) and *not* (N). a, Hybridization crosses. **b**, First backcrosses. **c**, Second backcrosses. **d-g**, Inbreeding crosses. Single pair matings were conducted for crosses a-c, mass matings for crosses d-g. Z-strain inherited alleles are orange and E-strain inherited alleles are blue. Females are the heterogametic sex (ZW) and males are the homogametic sex (ZZ) in which crossing-over can occur. Curly brackets point to the offspring of a certain cross. Grey boxes show recombination types of individuals that were selected via PCR amplifications for further crossing. Dotted black boxes show fixed homozygote *Resp*-recombinant lines. BC, backcross; IB, inbred cross; x, mating; f, female; m, male.

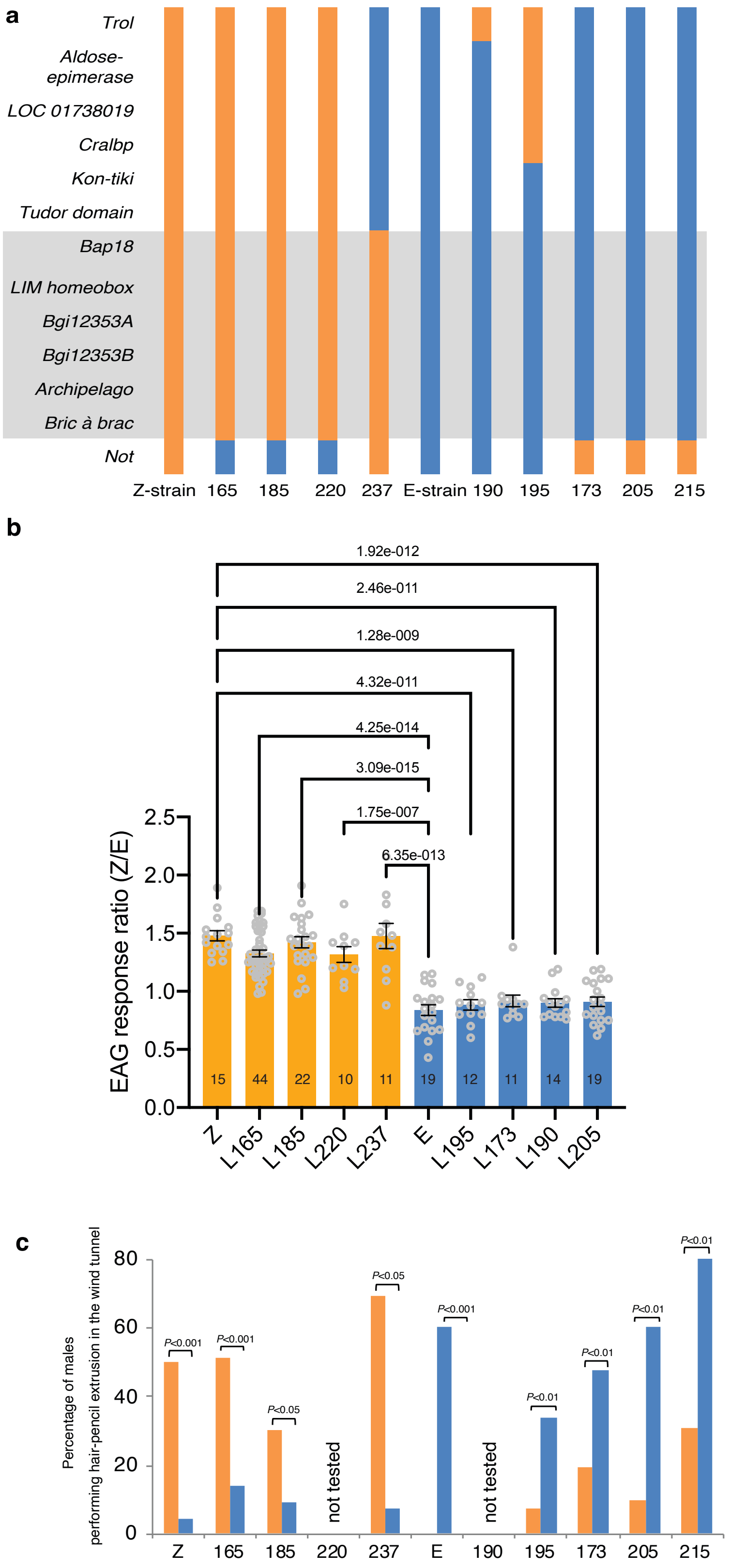

Supplementary Figure 2 | Genetic and behavioral analysis of *Resp*-recombinant lines. a, Genetic architecture of different *Resp*-recombinant inbred lines with crossovers between flanking genes *trol* and *not*. The bars represent the Z chromosome with gene regions identified within the *Resp* region (as reported by Koutroumpa, et al. [1]. Gene regions originating from the Z-strain are orange, those from the E-strain are blue. The grey box shows the chromosomal region with origin (E or Z) that correlates with the phenotypes of shown in b and c (E-responding or Z-responding). **b**, Electroantennogram (EAG) responses of pure strain and *Resp*-recombinant males. Bar heights show the mean ± SEM response to pure Z11-14:OAc divided by the mean response to pure E11-14:OAc. Z-strain and Z-like responses are shown in orange, E-strain and E-like reponses are shown in blue. Sample sizes of measured animals are shown on bars. *P* values report statistically significant comparisons with E-strain and Z-strain males, using two-sided Tukey's HSD post-hoc tests after an ANOVA (F = 30.11, df = 9, $P = 1.04 \times 10^{-30}$). **c**, Wind tunnel responses of pure strain and *Resp*-recombinant males to strain-specific pheromone lures. Orange bars represent the response to the Z-strain pheromone lure (97% Z-isomer, 3% E-isomer), blue bars show attraction to the E-strain lure (1% Z-isomer, 99% E-isomer). Data were analyzed with Chi-squared tests. Sample sizes of measured animals are: Z-strain *n* = 44, L165 *n* = 78, L185 *n* = 43, L237 *n* = 13, E-strain *n* = 40, L195 *n* = 53, L173 *n* = 57, L205 *n* = 31, and L215 *n* = 36. Source data are provided as a Source Data file.

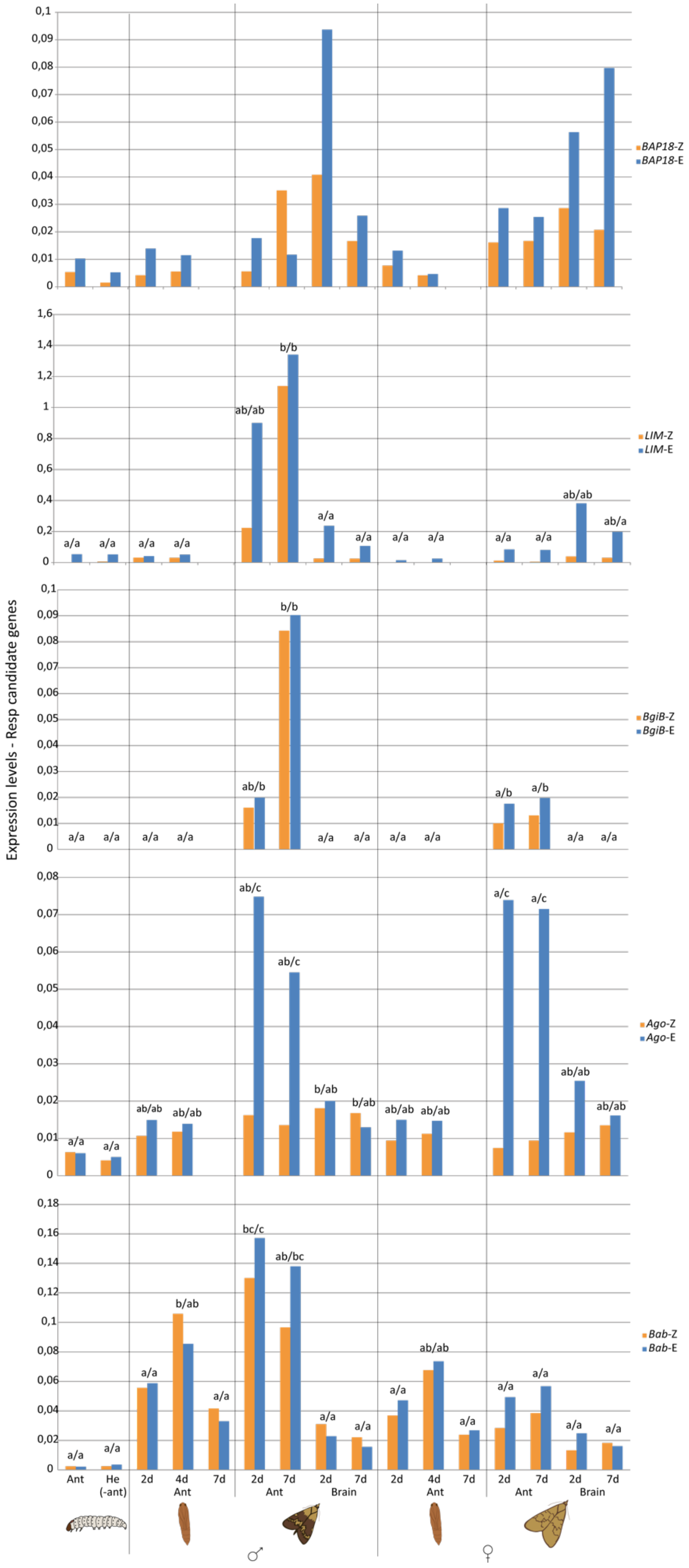

Supplementary Figure 3 | Transcript expression of the five *Resp* candidate genes. Mean expression values are normalized using *rpl8* as reference gene (ΔCT analysis). Orange bars represent Z-strain and blue bars E-strain. Ant, Antennae; He, Head; -ant, without antennae; d, days. A one-way ANOVA per candidate gene yielded significant variation among all strain by tissue by life-stage combinations. Letters indicate significant differences in two-sided Tukey's HSD post-hoc tests ($P < 0.05$), where a slash '/' separates results of strains (Z/E). Significance test values for 27 degrees of difference (31 degrees of difference for *bab* gene), after a Benjamini-Hochberg multiple-test correction, gave the following values per gene: *BAP18* $F = 1.76$, $P = 0.0373$; *BgiB* $F = 5.41$, $P = 8.47\text{e-}08$; *LIM* $F = 4.65$, $P = 7.48\text{e-}07$; *Ago* $F = 13.82$, $P = 8.02\text{e-}16$; *bab* $F = 29.65$, $P = 8.02\text{e-}16$. Source data are provided as a Source Data file.

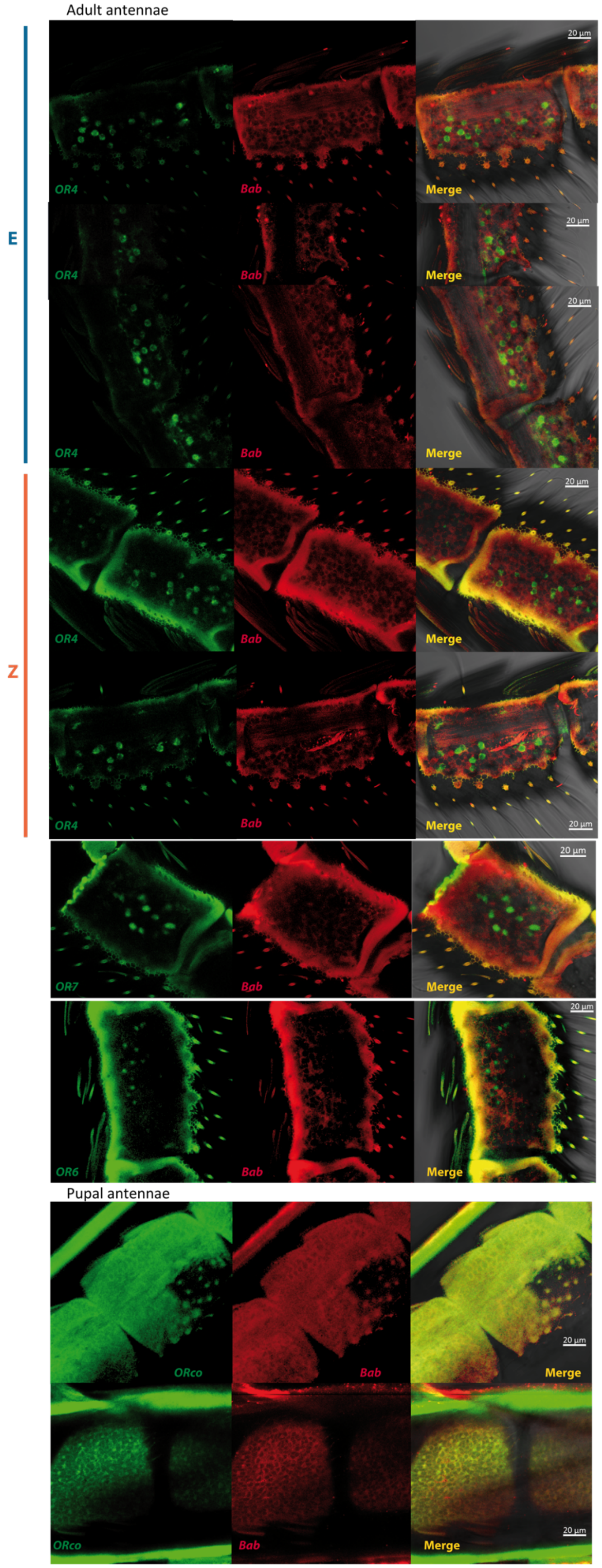

Supplementary Figure 4 | Additional panels of *in situ* hybridization of *bab* (red: digoxigenin) and odorant receptors (*OR4*, *OR6*, *OR7*) and co-receptor (*ORco*) (green: biotin) antennal transcripts. In merged images on the right, yellow indicates overlapping of the two signals. Successful demonstrations in pupal antennae were obtained 4 times for *bab*/*Orco* combination. Successful demonstrations in adult antennae were obtained 22 times for *bab*/*OR4* combination, 1 time for *bab*/*OR6* combination, 8 times for *bab*/*OR7* combination and 7 times for *bab*/*ORco* combination. The scale bar is 20 µm.

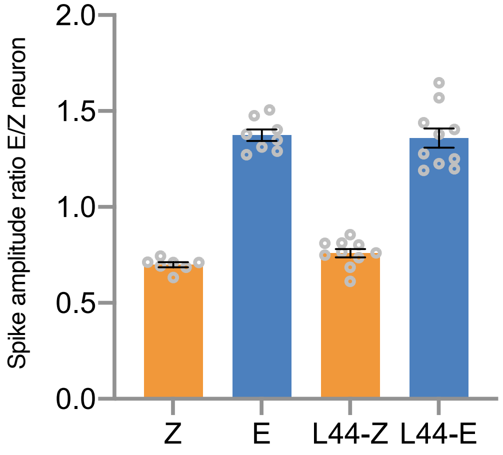

Supplementary Figure 5 | Single-sensillum recordings (SSRs) of pure strain and *bab*-recombinant males. Line L44-E has *bab* exon 1 to exon 2 from the E-strain and line L44-Z has exon 1 to exon 1.5 from the Z-strain (Fig. 2a). Heights of the bars show the mean ± SEM spike amplitude responding to pure E11-14:OAc divided by the mean spike amplitude responding to pure Z11-14:OAc. Z-strain and Z-like responses are shown in orange, E-strain and E-like reponses are shown in blue. Sample sizes of measured animals are Z-strain n = 7, E-strain n = 8, L44-E n = 10, L44-Z n = 10. Source data are provided as a Source Data file.

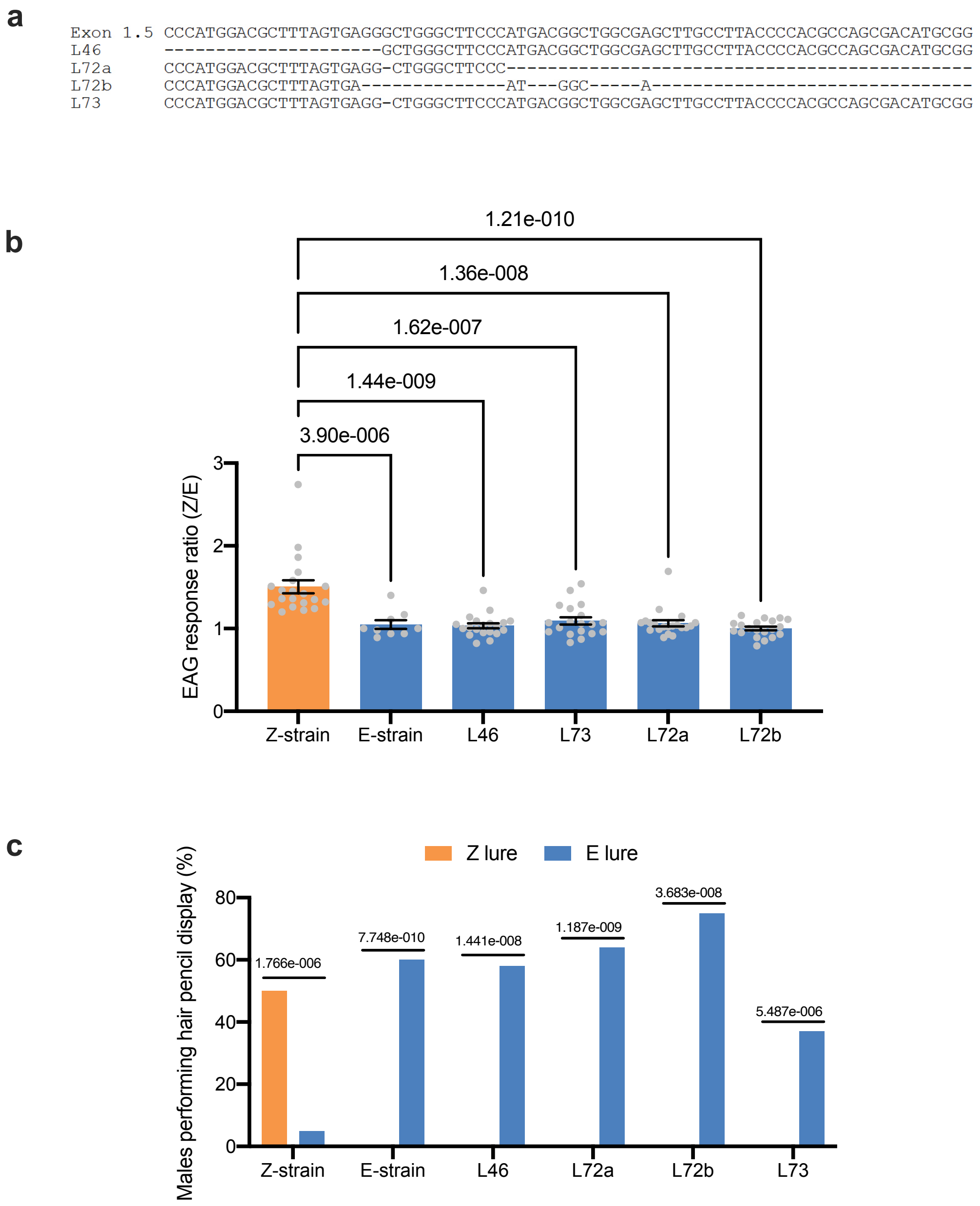

Supplementary Figure 6│ Genetic and behavioral analysis of CRISPR lines. a, CRISPR/Cas9-induced mutations of *bab* exon 1.5 in four mutant animals. **b,** Electroantennogram (EAG) responses of pure strain and CRISPR males. Bars show mean ± SEM relative response to pure Z11-14:OAc divided by the mean response to pure E11-14:OAc. Z-strain and Z-like responses are shown in orange, E-strain and E-like reponses are shown in blue. Sample sizes of animals measured are Z-strain n = 20, E-strain n = 9, L46 n = 20, L72a n = 20, L72b n = 20, L73 n = 19. P values report results of two-sided Tukey's HSD post-hoc tests after an ANOVA (F = 15.35, df = 5, $P = 2.94 \times 10^{-11}$). **c**, Wind tunnel responses of pure strain CRISPR males to strain-specific pheromone lures. Orange bars depict response to the Z-strain pheromone lure (97% Z-isomer, 3% E-isomer), blue bars depict response to the E-strain lure (1% Z-isomer, 99% E-isomer). Sample sizes of the number of animals studied are Z-strain n = 44, E-strain n = 40, L46 n = 36, L72a n = 36, L72b n = 24, and L73 n = 43. P values report results of two-sided Fisher's exact tests. Source data are provided as a Source Data file.

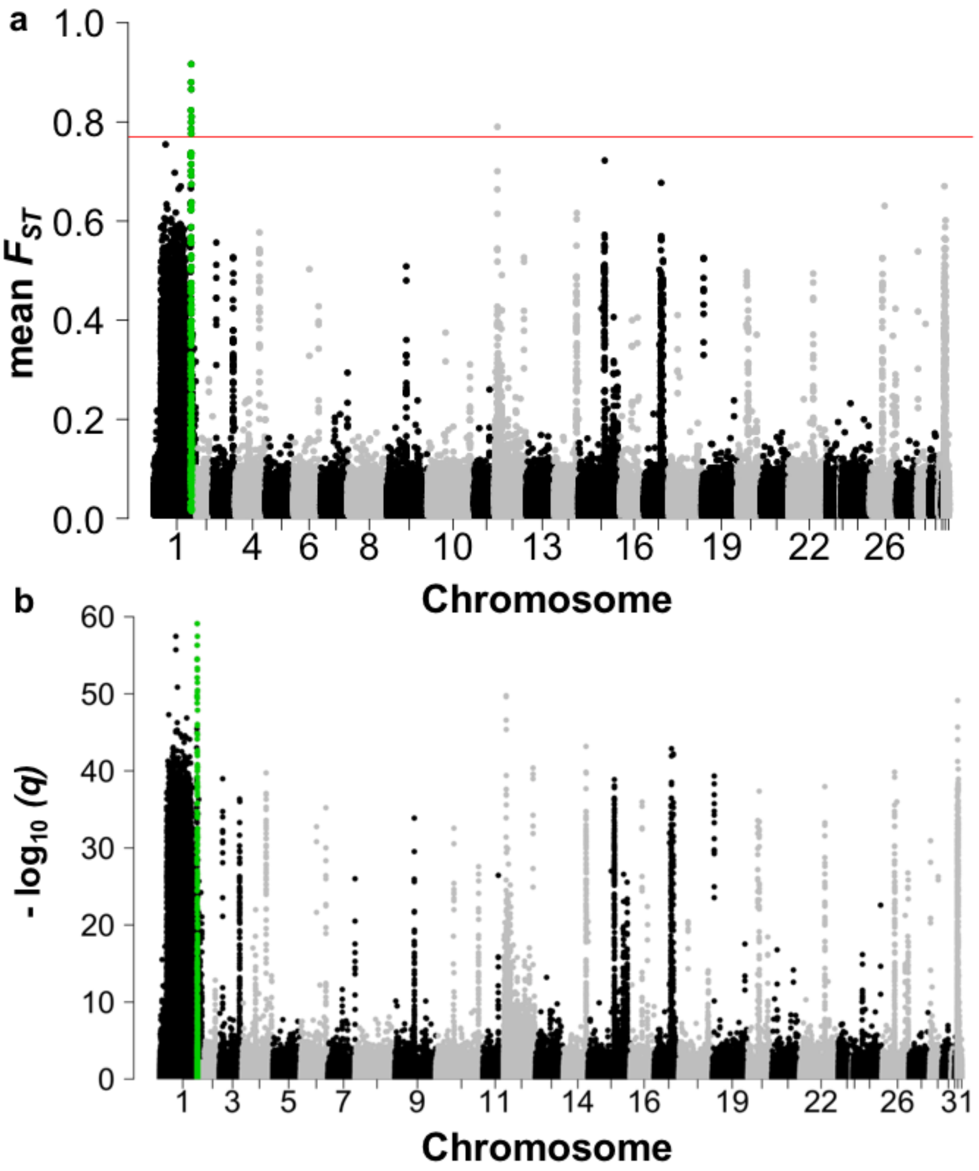

Supplementary Figure 7 | Comparison of sympatric males between pheromone trapped field collections. a, Mean F_{ST} between E and Z trapped males from 3 sites (1 kb windows; pool-seq of 25-41 males per trap per site; 97 Z-strain males, 106 E-strain males total) plotted across the *O. nubilalis* genome (chromosomes 1-31, unplaced scaffolds included at the end). b, False discovery rate (FDR) *q* values from Cochran-Mantel-Haenzel tests (negative log transformed) of allele frequency differences between E-Z pairs across sites (biallelic SNPs, passing Woolf heterogeneity test). *bab* gene region on chromosome 1 (Z) in green, red line represents the 99% quantile for regions physically linked to the pheromone locus (*pgFAR*, chr. 12) and *Resp* QTL.

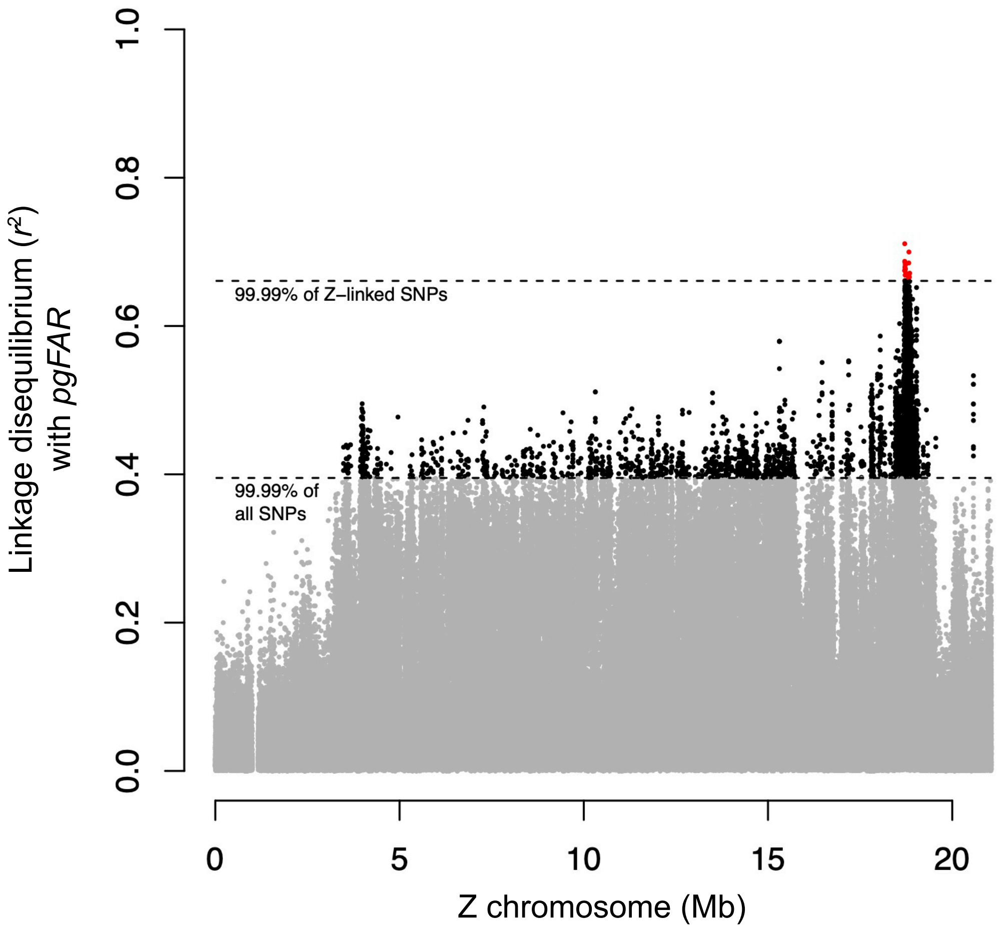

Supplementary Figure 8│ Linkage disequilibrium (r^2) between the autosomal gene controlling female pheromone blend (*pgFAR*) and the Z (sex) chromosome (Mb) for males attracted to Z or E sex pheromone. Of 31 amino-acid changing mutations at *pgFAR*, the one showing maximum r^2 with each Z-linked polymorphism (281,385 SNPs) is plotted. Red points depict r^2 values falling above the 99.99th percentile ($r^2 \approx 0.66$, top dashed line, 26 SNPs) of variants across the ~21 Mb Z chromosome. Black points (n = 3,668 SNPs) fall above the genome-wide 99.99th percentile ($r^2 \approx 0.4$, bottom dashed line) for variants separated by at least 1 Mb or on different chromosomes (1,350 SNPs considered).

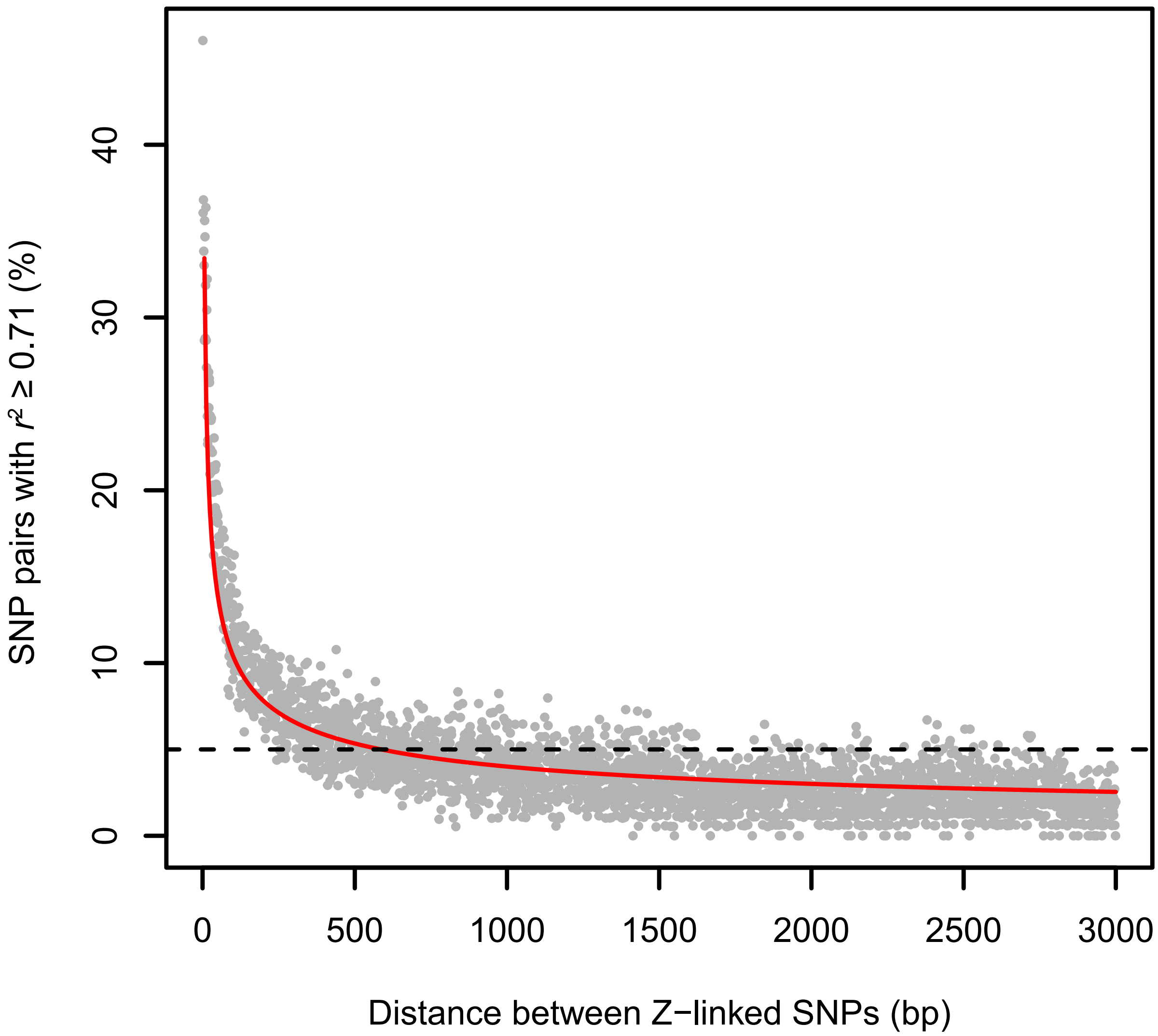

Supplementary Figure 9│ Percentage of sites separated by ≤ 3 kb of physical distance on the Z chromosome showing LD values ≥ the maximum LD observed between *pgFAR* and *bab* (r^2 = 0.71). LD values were calculated from 15 kb regions haphazardly sampled across all Z scaffolds ≥ 50 kb (*n* = 57). Depicted are LD values of 573,375 SNP pairs occurring within 1 bp–3,000 bp of physical distance. The red line is a best fit power curve (a = 70.36, $P < 2.2e-16$; b = -0.41, $P < 2.2e-16$; RSE = 1.64 (2,998 d.f.)). Less than half (46%) of SNPs separated by 1 base pair of physical distance on the Z chromosome had LD values as extreme as that observed between the physically unlinked loci *pgFAR* and *bab*. Fewer than 5% of SNPs separated by ~600 bp or more on the Z chromosome had r^2 values as extreme (dashed line).

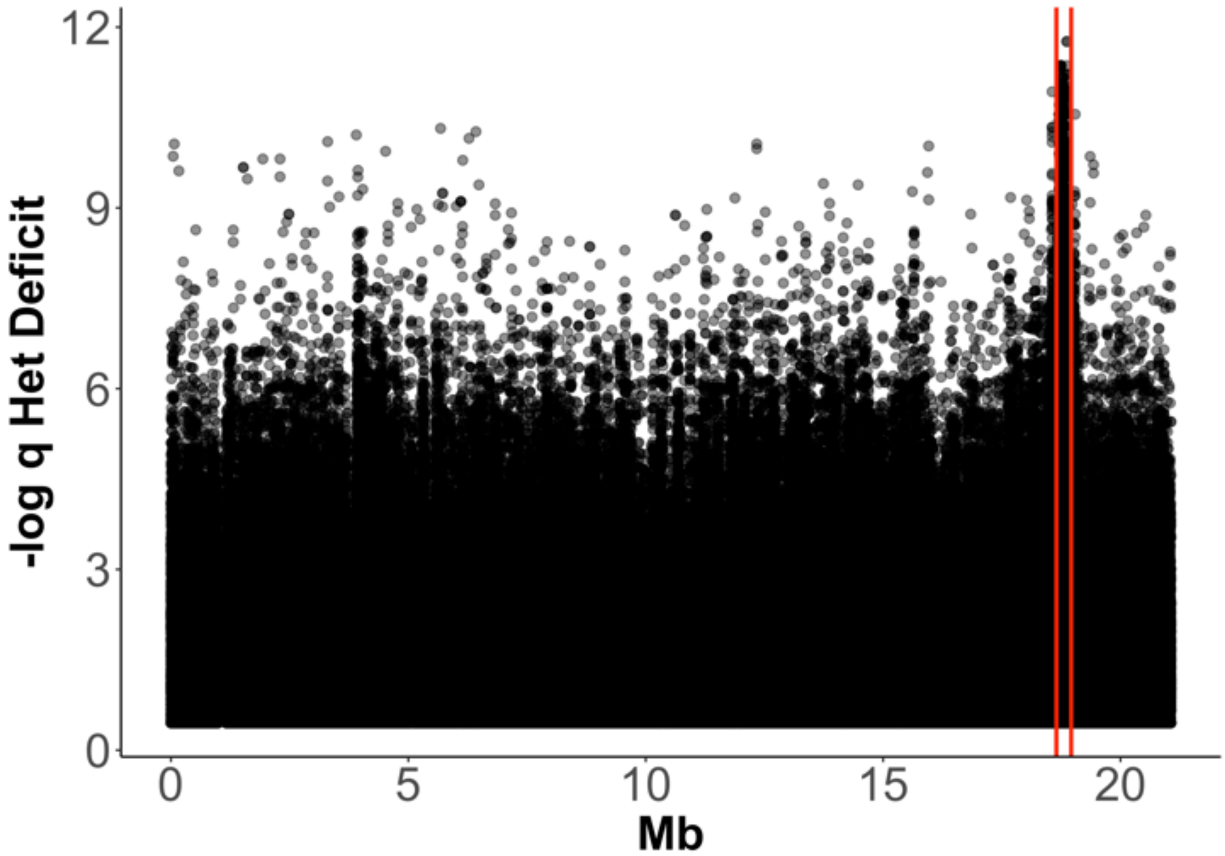

Supplementary Figure 10│ Heterozygosity deficit across the Z chromosome. -log10 FDR corrected *P*-values (*q*) for a test of heterozygote deficiency across the Z chromosome. The boundaries of *bab* are labeled with red lines.

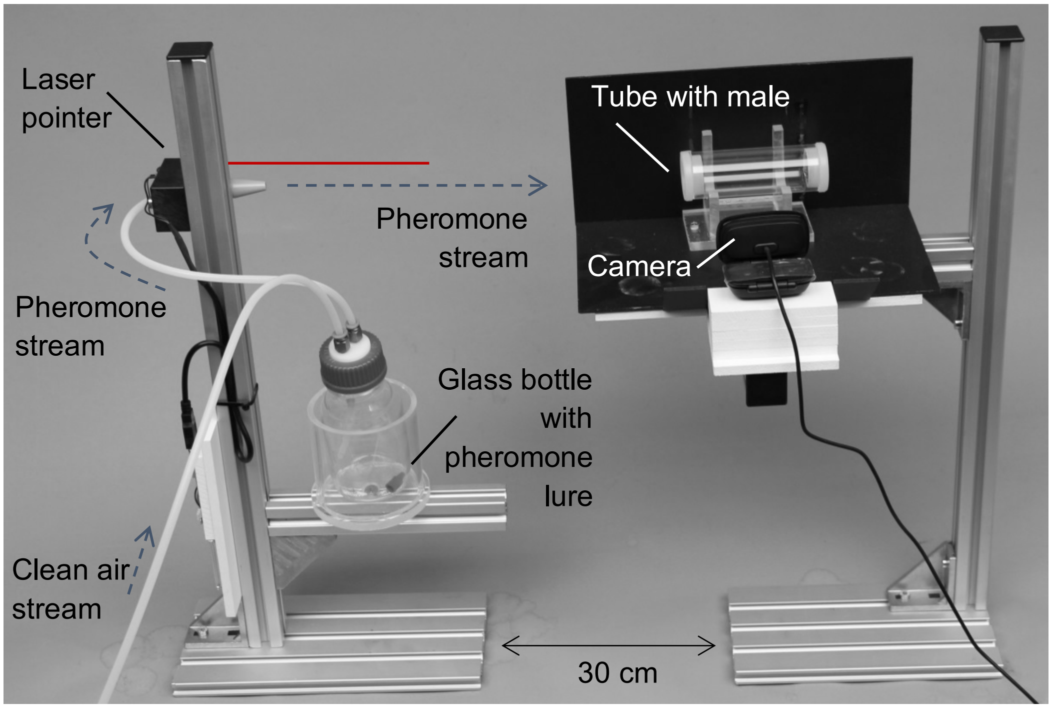

Supplementary Figure 11 | Experimental setup to observe male attraction to strain-specific pheromone lures in the wind tunnel. One stand (right) that contained a platform with a male tube was placed downwind at the end of the wind tunnel. Males were observed with an infrared camera connected to a PC. A second stand (left) was placed 30 cm in front of the male-stand and contained a pheromone bottle, which had two flexible pipes. One pipe was connected to compressed clean air, which streamed into the pheromone bottle when a foot pedal was pressed. The other pipe delivered the pheromone enriched air into the male tube. A laser pointer was used to adjust the left stand in front of the right stand so that the pheromone stream was exactly directed into the male tube.

Supplementary Table 1: PCR-based genetic analysis of *Resp*- and *bab1*-recombinant lines.

**PCR analysis**	**Primer No.**	**Location** [2]	**Forward Primer (5‘-3‘)**		**Reverse Primer (5‘-3‘)**		**PCR program**	**Size PCR product**
trol/not analysis of *Resp*-lines	1	*trol*<br>intron 27	Trol-Fwd16	CACGGACTTCG TCATGGAG	Trol-Rev17	TAGATGGGTCG CCGTAGGT	94°C 3 min =P1<br>94°C 50 sec<br>56°C 45 sec 30x<br>72°C 90 sec<br>72°C 10 min	Z-strain: 740 bp[1]<br>E-strain: 685 bp ($E_{allele1}$)<br>or 1100 bp ($E_{allele2}$)
	2	*not*<br>intron 13	Not-Fwd42	CGACTTCGACT ATGAGGGAAG	Not-Rev43	GAGGCAGCACG AGAAATAGTG		Z-strain: 1180 bp<br>E-strain: 1120 bp
ago/not analysis of *bab*-lines	3	*ago-bab-ig*	bab-g408-F1	AACGCACAGTG TCGTCTGACT	bab-g408-R2	GGTGACGTGTG TATTGATCAGC	94°C 3 min =P2<br>94°C 50 sec<br>55°C 45 sec 30x<br>72°C 60 sec<br>72°C 10 min	Line 165: 412 bp<br>Line 205: 280 bp
	4	*not-bab-ig*	bab-g048k-F3	CCTGAATGATG AGCTCTTAGAC AC	bab-g048k-R4	CTCTTCGGACTT GCGATTGT		Line 165: 186 bp<br>Line 205: 107 bp
break-point analysis	2	*not*<br>intron 13	Not-Fwd42	CGACTTCGACT ATGAGGGAAG	Not-Rev43	GAGGCAGCACG AGAAATAGTG	P1	Line 165: 1180 bp<br>Line 205: 1120 bp

of all lines	5	*not-bab-ig*	bab-g048k-F1	CATTGAGTGACATGCCGAAG	bab-g048k-R2	TGTCTAAGAGCTCATCATTCAGGT	P2	Line 165: 122 bp<br>Line 205: 271 bp
	4	*not-bab-ig*	bab-g048k-F3	CCTGAATGATGAGCTCTTAGACAC	bab-g048k-R4	CTCTTCGGACTTGCGATTGT	P2	Line 165: 186 bp<br>Line 205: 107 bp
	6	*bab*<br>exon 1	bab-e1-F7	AACACCGTTTACGTTGCAGA	bab-e1-R4	CGGGAAAACTTTTACGAGCA	95°C 3 min =P3<br>95°C 15 sec<br>60°C 15 sec 35x<br>72°C 30 sec<br>72°C 1 min	Z- and E-strain: 735 bp<br>→strain identification via Sanger sequencing
	7	*bab*<br>intron 1	bab-g150k-F1	CCGCTGTTTGTGAGAGGTTT	bab-g150k-R2	ATCGCCTCCCTCGTTTATTC	P2	Line 165: 492 bp<br>Line 205: 255 bp
	8	*bab*<br>intron 1	bab-g245k-F1	CCGTAACCGAAACCGGTATAA	bab-g245k-R2	CGAAGTAACTGTATGACGGGTGTA	P2	Line 165: 370 bp<br>Line 205: no band
	9	*bab*<br>exon 1.5	bab-e15L-F2	GGCAATTACCGCGTATCAGT	bab-e15L-R5	AAGCCCAGCCCTCACTAAAG	94°C 3 min =P4<br>94°C 50 sec	Line 165: 1576 bp<br>Line 205: 1205 bp

							55°C 45 sec 40x<br>72°C 150 sec<br>72°C 10 min	
	10	*bab*<br>intron 1.5	bab-g381k-F1	TGCAAACAACCTGTCGAAAC	bab-g381k-R2	CCCGTAGCCAGTGTCTGAAG	P2	Line 165: 443 bp<br>Line 205: 307 bp
	11	*bab*<br>exon 3	bab-e03-F3	ACCAATACCAGCTGCAATCC	bab-e03-R4	CCCAGCATGTTGTGGACTC	94°C 3 min =P5<br>94°C 30 sec<br>55°C 30 sec 35x<br>72°C 60 sec<br>72°C 10 min	Z- and E-strain: 235 bp<br>→strain identification via Sanger sequencing
	3	*ago-bab-*ig	bab-g408-F1	AACGCACAGTGTCGTCTGACT	bab-g408-R2	GGTGACGTGTGTATTGATCAGC	P2	Line 165: 412 bp<br>Line 205: 280 bp
	12	*ago*<br>intron 7	bab-g479-F3	AATCTTGCTCAAGCAGTCTGG	bab-g479-R4	TGACACCAAACAAAGGTCCA	P2	Line 165: 411 bp<br>Line 205: 294 bp
	13	*bgi12353 A-LIM*-ig	bA-LIM-1-F1	CGTGAAGGGCAACAAACAC	bA-LIM-1-R2	CCGTAGCTAGAGGCGAGAGA	P2	Line 165: 254 bp<br>Line 205: 278 bp

	14		bA-LIM-2-F1	AGCGGTGAGCGAGGTATAAA	bA-LIM-2-R2	TGCCAAATGTTTGTCTCGAA	P2	Line 165: 354 bp<br>Line 205: 315 bp
	15		bA-LIM-3-F1	GGATTGAGCGTACCAACCAG	bA-LIM-3-R2	ATGTGTTCTCACGCAGATCG	P2	Line 165: 217 bp<br>Line 205: 243 bp
	16	*BAP18-Tudor domain-*ig	BAP28-kon-F1	ACTGTTACCGCCTTCACTGC	BAP28-kon-R2	CAAAGGTCAAACAAGGTCACG	P2	Line 165: 175 bp<br>Line 205: no band
	17		BAP28-kon-F1	ACTGTTACCGCCTTCACTGC	BAP28-kon-R4	TCGAGGGTCTATGTGCGACT	P2	Line 165: no band<br>Line 205: 249 bp
	18		BAP18-kon-F3	TGCGTTTGAAAGACAAATTAGTACA	BAP18-kon-R4	TCGAGGGTCTATGTGCGACT	P2	Line 165: 329 bp<br>Line 205: no band
	19	*kon-Cralbp-*ig	kon-Cralbp1-F1	CGAAACCGCGTATTCAATTT	kon-Cralbp1-R2	TGTGCCATGCTAAGAACGAC	P2	Line 165: 377 bp<br>Line 205: 490 bp
	20	*Cralbp-Aldose-1 epimerase*-ig	Cralb-AldEpox1-F1	AGGCTGAGTTGCACCATCTT	Cralb-AldEpox1- R2	AGCTTCACCGTCGTTCAAAG	P2	Line 165: 413 bp<br>Line 205: 347 bp
	1	*trol*<br>intron 27	Trol-Fwd16	CACGGACTTCGTCATGGAG	Trol-Rev17	TAGATGGGTCGCCGTAGGT	P1	Z-strain: 740 bp<br>E-strain: 685 bp ($E_{allele1}$)

								or 1100 bp ($E_{allele2}$)

[1] For the *trol* gene, the Z-strain was selected before crossing experiments started for the genotype that exhibited a band of 740 bp.
[2] ig=intergenetic region

Supplementary Table 2: QPCR primers and respective annealing temperatures (Ta).

Gene name abbreviatio	Primer name	Sequence	PCR product size	Ta (°C)
Bab1	bab-e03-F3	ACCAATACCAGCTGCAATCC	217	60
	bab-e03-R5	CGGTTGGCATACAGCACGAA		
Ago	Ago-qPCR-F1	AATAGGGTGGTGTCCGGATC	134	60
	Ago-qPCR-R2	GACACCACCACTTTTCCGTC		
LIM	OnubLIM-qF1	AAGCCTCCTTCGAAGTCAGC	217	58
	OnubLIM-qR2	CTTGACGGTGACTCCTGCTT		
Bap18	OnubBap18-qF1	TCGTTCGCAATGCTGACTCT	212	59
	OnubBap18-qR2	AGGGGATTAGACAACAGCG		
BgiA	OnubBgiA-qF3	CCCCTGATATGTCTCGTGCC	185	60
	OnubBgiA-qR4	GCGCTGAATGGGTCCAAGTA		
BgiB	OnubBgiB-qF3	TACCCTGCTGATCAATGCCC	249	60
	OnubBgiB-qR4	ACAATACCACACCAACGCCA		
Orco	OL1-up	TTTACCGTTCCTTGGGTCTG	145	60
	OL1-do	GCCCTGAGCTTTCACTTTTG		
OnubOR6	ZOR6-up	TGGGCCTTTATTAGCCTTCA	184	60
	ZOR6-down	CAGCTTTTCCGTCTCTGACC		
GAPDH	OnubGAPDH-F1	CTTCGGCTCACTTGGAAGGT	166	59
	OnubGAPDH-R2	ACTTTAGCCAGAGGAGCAA		
18S	Onub18S-qF1	GCGTTGCTGGGAAGTTGAC	189	58
	Onub18S-qR2	ACGAGACCTTCCTTCGATCA		
rpL8	rpl8-up	ATGCCTGTGGGTGCTATGC	189	60
	rpl8-do	TGCCTCTGTTGCTTGATGGT		

Supplementary Table 3: SgRNA sequences and their initial concentrations.

Guide name	Guide Sequence	Concentration (µM)
1041	ATAAGATAAAATACCGAATGGGG	54
1042	TGTTTTTTGTATTCCCCATTCGG	16
1043	AATCTGGCTATTATACAAGAAGG	86
1044	ATGGACGCTTTAGTGAGGGCTGG	66
1045	TGGACGCTTTAGTGAGGGCTGGG	34
1046	GAGGGCTGGGCTTCCCATGACGG	59
1047	AAGGGTTAATAAGCATTCCGGGG	53
1048	CGCTTGATTGAATTTCTGAATGG	59
1049	TAATACTAATCTTGGCAGTGTGG	30

Supplementary Table 4: Field trapping of *O. nubilalis* males in North America

	**Number of individuals pooled by *pgFAR* genotype**		**Individuals resequenced by pheromone trap**			
**Site**	**EE**	**ZZ**	**E trap**	**Z trap**	**Latitude**	**Longitude**
Bellona, NY, USA	31	25	0	0	42°45' N	77°01' W
Landisville, PA, USA	41	39	16	16	40°02' N	76°18' W
Rockspring, PA, USA	34	33	15	15	40° 42' N	77°57' W

Supplementary Methods

PCR-based genotyping of recombinant lines

DNA extractions of single adult legs were performed as described[2]. To determine recombination events in the *Resp*-recombinant lines, PCRs were conducted by mixing 3 µl DNA with 11.90 µl ddH_2O, 2 µl 10x Taq buffer, 2 µl 2.5 mM dNTPs, 1 µl 10 mM primer mix (Supplementary Table 1, primer No. 1 and 2), and 0.1 µl or 0.2 µl mi-Taq polymerase (Metabion, Planegg, Germany) for the *trol* and *not* amplification, respectively. Size polymorphisms of the amplification products (Supplementary Table 1, see size PCR product) were visualized on a 2% agarose gel using Gene Ruler DNA ladder Mix (Thermo Fisher Scientific, Waltham, Massachusetts, USA) for size comparison. For the *bab*-recombinant lines, PCR reactions were conducted as described above using 0.1 µl mi-Taq polymerase for amplification of the intergenic region between *bab* and *ago* (Supplementary Table 1, primer No. 3) and 0.2 µl mi-Taq for amplification of the region between *bab* and *not* (Supplementary Table 1, primer No. 4). Size polymorphisms were visualized on a 1% agarose gel.

Breakpoint analysis of recombinant lines

After genome sequencing of lines L165 and L205, primers were designed (which amplified line-specific size polymorphisms) and used to narrow down the breakpoint within all *Resp*-recombinant lines. A total of 18 primer combinations (Supplementary Table 1, primer No. 1-5, No. 7-10, No. 12-20) spanning the region between *trol* and *not* were tested. PCRs were conducted as described above using 0.2 µl mi-Taq polymerase and size polymorphisms were visualized on a 2% agarose gel (Supplementary Table 1). For breakpoint analysis of the *bab*-recombinant lines, 5

primer combinations (Supplementary Table 1, primer No. 3-4, 8-10) were used to screen for size polymorphism, and products of another 2 primer combinations (Supplementary Table 1, primer No. 6, 11) were sequenced to identify diagnostic SNPs in *bab* exon 1 and exon 3. Due to high GC-content of *bab* exon 1, PCR amplifications were performed with KAPA2G Robust PCR Kit (Kapa Biosystems, Roche Company, Basel, Switzerland). Three µl DNA diluted in 7 µl deionized water were pre-denatured at 98°C for 5 min and then mixed with 5.8 µl deionized water, 5 µl KAPA2G GC buffer, 0.5 µl dNTPs, 1 µl Dimethyl sulfoxide, 0.2 µl KAPA2G Robust DNA Polymerase, and 2.5 µl primer mix (Supplementary Table 1, primer No. 6). The PCR products were cleaned with ZR-96 DNA Clean-up Kit (Zymo Research, California, USA) and Sanger-sequenced at the MPI-CE. PCR reactions of *bab* exon 3 were conducted as described above (PCR-based genotyping of recombinant lines) and products were visualized on a 1% agarose gel, cleaned with innuPREP Gel Extraction Kit (Analytik Jena AG, Jena, Germany), and Sanger-sequenced at the MPI-CE.

Phenotyping with electrophysiological recordings

Electroantennogram (EAG) recordings of 0-4 day-old males were performed at SLU in Sweden (Resp-recombinant lines) and at MPI-CE in Germany (*bab*-recombinant and CRISPR lines). Male heads were crushed to remove mechanical disturbances. Heads were placed on one side of a fork-shaped silver electrode holder (Syntech, Buchebach, Germany) with the tip of the antenna on the other side. An electrolyte gel (Blågel, Cefar, Malmö, Sweden or Spectra 360 Electrode Gel, Parker Laboratories, Fairfield, New Jersey, USA) on both electrodes provided conductivity and prevented dehydration. Ten ng of Z11-14:OAc or E11-14:OAc (Pherobank, Wijk bij Duurstede, Netherlands) was pipetted onto a 12.7 mm diameter round filter paper disc (Schleicher & Schnell GmbH, Dassel, Germany) and placed into a glass Pasteur pipette. Control

pipettes contained a filter paper with 10 µl hexane alone. A stimulus controller (CS-55, Syntech, Buchebach, Germany) produced a flow of 1 liter/min charcoal-filtered air over the antennae. Antennae were stimulated with 0.5 s odor puffs (block pulses) using the following sequence: 1) hexane control, 2) E11-14:OAc, and 3) Z11-14:OAc, with an inter-stimulus interval of 30 s. EAG signals were analyzed with Syntech software (GC/EAD 32 version 4.3, and Autospike Version 3.9, Syntech). In the statistical analysis, we calculated the ratio of (Z11-14:OAc-control)/(E11-14:OAc-control) and analyzed the data in R[3] with a one-way analysis of variance (ANOVA) followed by a two-sided Tukey's HSD post hoc test.

Phenotyping using single sensillum recordings (SSR) was done at SLU (Sweden) on *bab*-recombinant lines using 2-6 day-old males, using methods previously described[4]. Briefly, males were constrained in 1 ml pipette tips with both antennae extruding from the cut tip. The constrained animal was placed on a bed of dental wax and antennae were gently immobilized on a coverslip with insect glue (Csalomon, Hungary). A tungsten electrode inserted into the abdomen served as the reference electrode. The preparation was placed under a light microscope (Olympus BX 51W1) at 500x magnification. An electrolytically-sharpened electrode was placed at the base of a sensillum and responses to air puffs (5 ml/s) containing either one of the two pheromone components or clean air. The air puffs led via a Pasteur pipette with a filter paper cartridge, loaded with typically 100 ng of one of the pheromone components, into a 500 ml/min humidified and charcoal filtered airstream over the preparation. Stimulations were randomly presented at minimally 30 s apart. Responses were collected and spike amplitude was analyzed using Autospike software (Syntech, Buchebach, Germany). To verify consistency in spike amplitude, several recordings were done per animal (which was 100%). A single trace per animal was used for further analyses.

Genetic analysis of bab exon 1

bab exon 1 was amplified and sequenced from 10-36 males per *Resp*- and *bab*-recombinant line, ~100 Z- and E-strain males of European laboratory populations, ~50 field trapped individuals from the United States, and ~50 Z- and E-strain males of laboratory populations from the United States. US laboratory populations consisting of univoltine Z and bivoltine E corn borers (>500 males and >500 females of each) were collected in 1994 from Bouckville, NY (42.8892°N, 75.5513°W) and in 1996 from Geneva, NY (42.8680°N, 76.9856°W), respectively. At both sites, fifth instar diapausing larvae or pupae were taken from stalks of *Zea mays*. Breeding colonies were reared at 26°C and 16:8 LD photoperiod. Larvae were fed a standard artificial European corn borer diet (Southland Products, Lake Village, AR, USA) and populations were reared en masse (>100 breeding pairs). All sequenced males were previously phenotyped with EAG or wind tunnel assays or with pheromone trapping. PCR and sequencing was performed as described above (see breakpoint analysis).

Supplementary References

1 Koutroumpa, F. A., Groot, A. T., Dekker, T. & Heckel, D. G. Genetic mapping of male pheromone response in the European corn borer identifies candidate genes regulating neurogenesis. *P Natl Acad Sci USA* **113**, E6401-E6408, doi:10.1073/pnas.1610515113 (2016).

2 Haenniger, S. *et al.* Sexual communication of *Spodoptera frugiperda* from West Africa: Adaptation of an invasive species and implications for pest management. *Sci Rep-Uk* **10**, doi:ARTN 2892 10.1038/s41598-020-59708-7 (2020).

3 R: A Language and Environment for Statistical Computing (R Foundation for Statistical Computing, Vienna, Austria., 2019).

4 Kárpáti, Z., Tasin, M., Carde, R. T. & Dekker, T. Early quality assessment lessens pheromone specificity in a moth. *P Natl Acad Sci USA* **110**, 7377-7382, doi:10.1073/pnas.1216145110 (2013).
