# Supplementary material for: bric à brac controls sex pheromone choice by male European corn borer moths
Source: Nat Commun. 2021 May 14;12:2818. doi: 10.1038/s41467-021-23026-x (PMC8121916; doi:10.1038/s41467-021-23026-x)
Supplement: Supplementary file 2 — Reporting Summary [file 41467_2021_23026_MOESM2_ESM.pdf]

nature research

Corresponding author(s): Erik B. Dopman; David Heckel

Last updated by author(s): Mar 8, 2021

Reporting Summary

Nature Research wishes to improve the reproducibility of the work that we publish. This form provides structure for consistency and transparency in reporting. For further information on Nature Research policies, see our Editorial Policies and the Editorial Policy Checklist.

Statistics

For all statistical analyses, confirm that the following items are present in the figure legend, table legend, main text, or Methods section.

n/a	Confirmed	
☐	☒	The exact sample size (*n*) for each experimental group/condition, given as a discrete number and unit of measurement
☐	☒	A statement on whether measurements were taken from distinct samples or whether the same sample was measured repeatedly
☐	☒	The statistical test(s) used AND whether they are one- or two-sided<br>*Only common tests should be described solely by name; describe more complex techniques in the Methods section.*
☐	☒	A description of all covariates tested
☐	☒	A description of any assumptions or corrections, such as tests of normality and adjustment for multiple comparisons
☐	☒	A full description of the statistical parameters including central tendency (e.g. means) or other basic estimates (e.g. regression coefficient) AND variation (e.g. standard deviation) or associated estimates of uncertainty (e.g. confidence intervals)
☐	☒	For null hypothesis testing, the test statistic (e.g. *F*, *t*, *r*) with confidence intervals, effect sizes, degrees of freedom and *P* value noted<br>*Give P values as exact values whenever suitable.*
☐	☒	For Bayesian analysis, information on the choice of priors and Markov chain Monte Carlo settings
☒	☐	For hierarchical and complex designs, identification of the appropriate level for tests and full reporting of outcomes
☐	☒	Estimates of effect sizes (e.g. Cohen's *d*, Pearson's *r*), indicating how they were calculated

Our web collection on statistics for biologists contains articles on many of the points above.

Software and code

Policy information about availability of computer code

Data collection	CRISPOR gRNA design tool v. 4.98, GC/EAD 32 v. 4.3, Autospike v. 3.9,
Data analysis	PacBio SMRT, SEQUENCHER™ 4.7, BayPass v.2.1, Beagle v.5, Bowtie2 v.2.2.3, bwa v.0.7.9a, Delly2, GATK v.3.7, ggplot2, Picardtools v.2.8.0, Popoolation2 v.2.2.1, Samtools v.1.18, vcftools v.0.1.12, CLC Genomics Workbench v.10.1, R v.3.6.1, Trinity v.7.17.14, Powerpoint v. 16.45, Adobe Illustrator v. 25.2, ggplot2, qqman, Trimmomatic v.35, fdrtool

For manuscripts utilizing custom algorithms or software that are central to the research but not yet described in published literature, software must be made available to editors and reviewers. We strongly encourage code deposition in a community repository (e.g. GitHub). See the Nature Research guidelines for submitting code & software for further information.

Data

Policy information about availability of data

All manuscripts must include a data availability statement. This statement should provide the following information, where applicable:

- Accession codes, unique identifiers, or web links for publicly available datasets
- A list of figures that have associated raw data
- A description of any restrictions on data availability

Data for all figures are available as Source Data or are publicly available at data repositories. Genome data are available from GenBank under multiple BioProjects. Pooled resequencing data can be found at BioSample PRJNA361472 (Rockspring, PA, Landisville, PA) and BioSample PRJNA655940 (Bellona, NY). Individual resequencing data can be found at BioSample PRJNA540833 (Landisville, PA, Z trap) and BioSample PRJNA656178 (Landisville, PA, E trap; Rockspring, PA, E trap; and Rockspring, PA, Z trap). New RNA-seq data are available at Bioproject PRJNA704411. Confocal images under accession number S-BSST601 are available at BioStudies.

Field-specific reporting

Please select the one below that is the best fit for your research. If you are not sure, read the appropriate sections before making your selection.

☒ Life sciences ☐ Behavioural & social sciences ☐ Ecological, evolutionary & environmental sciences

For a reference copy of the document with all sections, see nature.com/documents/nr-reporting-summary-flat.pdf

Life sciences study design

All studies must disclose on these points even when the disclosure is negative.

Sample size	No statistical methods were used to predetermine sample size. Sample size was demonstrated in pilot and prior experiments to be sufficient to achieve desired outcomes according to standards of the field.
Data exclusions	No data were excluded.
Replication	qPCR repeated across two replicates gave similar amplification efficiencies. Four CRISPR lines with exon 1.5 mutations each gave similar behavioral and physiological results. Whole mount in situ hybridization were repeated with similar results in pupal antennae 4 times for bab/Orco combinations and in adult antennae 22 times for bab/OR4 combinations, 8 times for bab/OR7 combinations, and 7 times for bab/Orco combinations.
Randomization	Experimental groups were defined by the specific pheromone blend used by individuals. The presentation order of synthetic pheromone blend to individuals was randomized during processing of behavioral and physiological response.
Blinding	Samples were blindly processed with respect to strain for behavioral and physiological data collection.

Reporting for specific materials, systems and methods

We require information from authors about some types of materials, experimental systems and methods used in many studies. Here, indicate whether each material, system or method listed is relevant to your study. If you are not sure if a list item applies to your research, read the appropriate section before selecting a response.

Materials & experimental systems

n/a	Involved in the study	
☒	☐	Antibodies
☒	☐	Eukaryotic cell lines
☒	☐	Palaeontology and archaeology
☐	☒	Animals and other organisms
☒	☐	Human research participants
☒	☐	Clinical data
☒	☐	Dual use research of concern

Methods

n/a	Involved in the study	
☒	☐	ChIP-seq
☒	☐	Flow cytometry
☒	☐	MRI-based neuroimaging

Animals and other organisms

Policy information about studies involving animals; ARRIVE guidelines recommended for reporting animal research

Laboratory animals	All individuals were Ostrinia nubilalis, the European corn borer moth. The Z-strain colony derived from mixed sex cornfield-collected adults in Kéty town, county of Tolna, Hungary in 2004. The E-strain colony was established from mixed sex larvae extracted from maize stems collected by Magda Rak-Cizej of the Agriculture and Forestry Institute, Novo Mesto, Slovenia. RNA-seq was conducted on US laboratory colonies consisting of mixed sex Z and E larvae and pupae corn borers collected from cornfields in 1994 from Bouckville, NY, and in 1996 from Geneva, NY, respectively.
Wild animals	Pheromone trapping in the US was used to collect wild E- and Z- pheromone preferring male adults using Scentry Heliothis traps baited with synthetic E ("New York") and Z ("Iowa") lures (Scentry Biologicals, Billings, MO, USA). Traps were placed directly next to sweet corn fields and males were collected from each trap every 1-2 weeks and stored at -20°C. Lures were replaced every 2 weeks. Trapping of > 20 male adults from each E and Z trap was done at 3 sympatric sites between 2010-2012. Tissues were moved from -20°C within 3 months of collection to at -80°C for long-term storage.
Field-collected samples	The study did not involve laboratory work with field-collected animals.
Ethics oversight	No ethical approval or guidance was required to study this insect pest.

Note that full information on the approval of the study protocol must also be provided in the manuscript.
