# Supplementary material for: bric à brac controls sex pheromone choice by male European corn borer moths
Source: Nat Commun. 2021 May 14;12:2818. doi: 10.1038/s41467-021-23026-x (PMC8121916; doi:10.1038/s41467-021-23026-x)
Supplement: Supplementary file 3 — Description of Additional Supplementary Files [file 41467_2021_23026_MOESM3_ESM.pdf]

Description of Additional Supplementary Files

File Name: Supplementary Data 1
Description: **Nucleotide alignment of bab exon 1 of different O. nubilalis populations**. Sequences were obtained from gDNA extraction of males that were previously phenotyped in the wind tunnel or with EAG recordings. Orange framed populations exhibited the Z-strain phenotype, blue framed populations the E- strain phenotype. Red marked bases highlight polymorphisms between different populations. Z chromosome coordinates 18.96172 Mb to 18.96105 Mb (note: gene/exon runs backwards relative to Z chromosome orientation).

File Name: Supplementary Data 2
Description: **Baypass and FST results for bab gene region.** Bayes Factor (BF) and F_{ST} values for polymorphism (SNP, indel, SV) associated with pheromone trap along the bab region of the Z chromosome for n = 62 field caught males.
