# Supplementary material for: bric à brac controls sex pheromone choice by male European corn borer moths
Source: Nat Commun. 2021 May 14;12:2818. doi: 10.1038/s41467-021-23026-x (PMC8121916; doi:10.1038/s41467-021-23026-x)
Supplement: Supplementary file 4 — Supplementary Data 1 [file 41467_2021_23026_MOESM4_ESM.docx]

**Supplementary Data 1: Nucleotide alignment of *bab* exon 1 of different *O. nubilalis* populations.** Sequences were obtained from gDNA extraction of males that were previously phenotyped in the wind tunnel or with EAG recordings. Orange framed populations exhibited the Z-strain phenotype, blue framed populations the E-strain phenotype. Red marked bases highlight polymorphisms between different populations. Z chromosome coordinates 18.96172 Mb to 18.96105 Mb (note: gene/exon runs backwards relative to Z chromosome orientation).

10 20 30 40 50

....|....|....|....|....|....|....|....|....|....|

Z-strain_Europe lab (n=52) ATGCCCGCCGAGGAGGAACCCGCGGCGATGGAGAGCCGGGACGCCGATGG

Z-strain_America lab (n=29) ATGCCCGCCGAGGAGGAACCCGCGGCGATGGAGAGCCGGGACGCCGATGG

Z-strain_America field (n=27) ATGCCCGCCGAGGAGGAACCCGCGGCGATGGAGAGCCGGGACGCCGATGG

L165_Europe (n=23) ATGCCCGCCGAGGAGGAACCCGCGGCGATGGAGAGCCGGGACGCCGATGG

L185_Europe (n=20) ATGCCCGCCGAGGAGGAACCCGCGGCGATGGAGAGCCGGGACGCCGATGG

L220_Europe (n=10) ATGCCCGCCGAGGAGGAACCCGCGGCGATGGAGAGCCGGGACGCCGATGG

L237_Europe (n=14) ATGCCCGCCGAGGAGGAACCCGCGGCGATGGAGAGCCGGGACGCCGATGG

L44-Z_Europe (n=22) ATGCCCGCCGAGGAGGAACCCGCGGCGATGGAGAGCCGGGACGCCGATGG

E-strain_Europe lab (n=59) ATGCCCGCCGAGGAGGAACCCGCGGCGATGGAGAGCCGGGACGCCGATGG

E-strain_America lab (n=21) ATGCCCGCCGAGGAGGAACCCGCGGCGATGGAGAGCCG**S**GACGCCGATGG

E-strain_America field (n=24) ATGCCCGCCGAGGAGGAACCCGCGGCGATGGAGAGCCG**S**GACGCCGATGG

L195_Europe (n=22) ATGCCCGCCGAGGAGGAACCCGCGGCGATGGAGAGCCGGGACGCCGATGG

L173__Europe (n=20) ATGCCCGCCGAGGAGGAACCCGCGGCGATGGAGAGCCGGGACGCCGATGG

L205_Europe (n=36) ATGCCCGCCGAGGAGGAACCCGCGGCGATGGAGAGCCGGGACGCCGATGG

L215_Europe (n=19) ATGCCCGCCGAGGAGGAACCCGCGGCGATGGAGAGCCGGGACGCCGATGG

L44-E_Europe (n=28) ATGCCCGCCGAGGAGGAACCCGCGGCGATGGAGAGCCGGGACGCCGATGG

60 70 80 90 100

....|....|....|....|....|....|....|....|....|....|

Z-strain_Europe lab (n=52) CTCGCCCCAACAATTCTGCCTGCGCTGGAACAACTACCAGAGCAACTTGG

Z-strain_America lab (n=29) CTCGCCCCAACAATTCTGCCTGCGCTGGAACAACTACCAGAGCAACTTGG

Z-strain_America field (n=27) CTCGCCCCAACAATTCTGCCTGCGCTGGAACAACTACCAGAGCAACTTGG

L165_Europe (n=23) CTCGCCCCAACAATTCTGCCTGCGCTGGAACAACTACCAGAGCAACTTGG

L185_Europe (n=20) CTCGCCCCAACAATTCTGCCTGCGCTGGAACAACTACCAGAGCAACTTGG

L220_Europe (n=10) CTCGCCCCAACAATTCTGCCTGCGCTGGAACAACTACCAGAGCAACTTGG

L237_Europe (n=14) CTCGCCCCAACAATTCTGCCTGCGCTGGAACAACTACCAGAGCAACTTGG

L44-Z_Europe (n=22) CTCGCCCCAACAATTCTGCCTGCGCTGGAACAACTACCAGAGCAACTTGG

E-strain_Europe lab (n=59) CTCGCCCCAACAATTCTGCCTGCGCTGGAACAACTACCAGAGCAACTTGG

E-strain_America lab (n=21) CTCGCCCCAACAATTCTGCCTGCGCTGGAACAACTACCAGAGCAACTTGG

E-strain_America field (n=24) CTCGCCCCAACAATTCTGCCTGCGCTGGAACAACTACCAGAGCAACTTGG

L195_Europe (n=22) CTCGCCCCAACAATTCTGCCTGCGCTGGAACAACTACCAGAGCAACTTGG

L173__Europe (n=20) CTCGCCCCAACAATTCTGCCTGCGCTGGAACAACTACCAGAGCAACTTGG

L205_Europe (n=36) CTCGCCCCAACAATTCTGCCTGCGCTGGAACAACTACCAGAGCAACTTGG

L215_Europe (n=19) CTCGCCCCAACAATTCTGCCTGCGCTGGAACAACTACCAGAGCAACTTGG

L44-E_Europe (n=28) CTCGCCCCAACAATTCTGCCTGCGCTGGAACAACTACCAGAGCAACTTGG

110 120 130 140 150

....|....|....|....|....|....|....|....|....|....|

Z-strain_Europe lab (n=52) CCAACTGTTTCGACCAGCTCCTCCAGACCGAGTCCTTCGTGGACGTCACG

Z-strain_America lab (n=29) CCAACTGTTTCGACCAGCTCCTCCAGACCGAGTCCTTCGTGGACGTCACG

Z-strain_America field (n=27) CCAACTGTTTCGACCAGCTCCTCCAGACCGAGTCCTTCGTGGACGTCACG

L165_Europe (n=23) CCAACTGTTTCGACCAGCTCCTCCAGACCGAGTCCTTCGTGGACGTCACG

L185_Europe (n=20) CCAACTGTTTCGACCAGCTCCTCCAGACCGAGTCCTTCGTGGACGTCACG

L220_Europe (n=10) CCAACTGTTTCGACCAGCTCCTCCAGACCGAGTCCTTCGTGGACGTCACG

L237_Europe (n=14) CCAACTGTTTCGACCAGCTCCTCCAGACCGAGTCCTTCGTGGACGTCACG

L44-Z_Europe (n=22) CCAACTGTTTCGACCAGCTCCTCCAGACCGAGTCCTTCGTGGACGTCACG

E-strain_Europe lab (n=59) CCAACTGTTTCGACCAGCTCCTCCAGACCGAGTCCTTCGTGGACGTCACG

E-strain_America lab (n=21) CCAACTGTTTCGACCAGCTCCTCCAGACCGAGTCCTTCGTGGACGTCACG

E-strain_America field (n=24) CCAACTGTTTCGACCAGCTCCTCCAGACCGAGTCCTTCGTGGACGTCACG

L195_Europe (n=22) CCAACTGTTTCGACCAGCTCCTCCAGACCGAGTCCTTCGTGGACGTCACG

L173__Europe (n=20) CCAACTGTTTCGACCAGCTCCTCCAGACCGAGTCCTTCGTGGACGTCACG

L205_Europe (n=36) CCAACTGTTTCGACCAGCTCCTCCAGACCGAGTCCTTCGTGGACGTCACG

L215_Europe (n=19) CCAACTGTTTCGACCAGCTCCTCCAGACCGAGTCCTTCGTGGACGTCACG

L44-E_Europe (n=28) CCAACTGTTTCGACCAGCTCCTCCAGACCGAGTCCTTCGTGGACGTCACG

160 170 180 190 200

....|....|....|....|....|....|....|....|....|....|

Z-strain_Europe lab (n=52) CTCGCGTGCGAGGGCCAGAGCCTGAAGGCGCACAAGGTGGTGCTGTCGGC

Z-strain_America lab (n=29) CTCGCGTGCGAGGGCCAGAGCCTGAAGGCGCACAAGGTGGTGCTGTCGGC

Z-strain_America field (n=27) CTCGCGTGCGAGGGCCAGAGCCTGAAGGCGCACAAGGTGGTGCTGTCGGC

L165_Europe (n=23) CTCGCGTGCGAGGGCCAGAGCCTGAAGGCGCACAAGGTGGTGCTGTCGGC

L185_Europe (n=20) CTCGCGTGCGAGGGCCAGAGCCTGAAGGCGCACAAGGTGGTGCTGTCGGC

L220_Europe (n=10) CTCGCGTGCGAGGGCCAGAGCCTGAAGGCGCACAAGGTGGTGCTGTCGGC

L237_Europe (n=14) CTCGCGTGCGAGGGCCAGAGCCTGAAGGCGCACAAGGTGGTGCTGTCGGC

L44-Z_Europe (n=22) CTCGCGTGCGAGGGCCAGAGCCTGAAGGCGCACAAGGTGGTGCTGTCGGC

E-strain_Europe lab (n=59) CTCGCGTGCGAGGGCCAGAGCCTGAAGGCGCACAAGGTGGTGCTGTCGGC

E-strain_America lab (n=21) CTCGCGTGCGAGGGCCAGAGCCTGAAGGCGCACAAGGTGGTGCTGTCGGC

E-strain_America field (n=24) CTCGCGTGCGAGGGCCAGAGCCTGAAGGCGCACAAGGTGGTGCTGTCGGC

L195_Europe (n=22) CTCGCGTGCGAGGGCCAGAGCCTGAAGGCGCACAAGGTGGTGCTGTCGGC

L173__Europe (n=20) CTCGCGTGCGAGGGCCAGAGCCTGAAGGCGCACAAGGTGGTGCTGTCGGC

L205_Europe (n=36) CTCGCGTGCGAGGGCCAGAGCCTGAAGGCGCACAAGGTGGTGCTGTCGGC

L215_Europe (n=19) CTCGCGTGCGAGGGCCAGAGCCTGAAGGCGCACAAGGTGGTGCTGTCGGC

L44-E_Europe (n=28) CTCGCGTGCGAGGGCCAGAGCCTGAAGGCGCACAAGGTGGTGCTGTCGGC

210 220 230 240 250

....|....|....|....|....|....|....|....|....|....|

Z-strain_Europe lab (n=52) GTGCAGCCCCTACTTCCAGTCGCTGTTCATGGACAACCCCTGCCGGCACC

Z-strain_America lab (n=29) GTGCAGCCCCTACTTCCAGTCGCTGTTCATGGACAACCCCTGCCGGCACC

Z-strain_America field (n=27) GTGCAGCCCCTACTTCCAGTCGCTGTTCATGGACAACCCCTGCCGGCACC

L165_Europe (n=23) GTGCAGCCCCTACTTCCAGTCGCTGTTCATGGACAACCCCTGCCGGCACC

L185_Europe (n=20) GTGCAGCCCCTACTTCCAGTCGCTGTTCATGGACAACCCCTGCCGGCACC

L220_Europe (n=10) GTGCAGCCCCTACTTCCAGTCGCTGTTCATGGACAACCCCTGCCGGCACC

L237_Europe (n=14) GTGCAGCCCCTACTTCCAGTCGCTGTTCATGGACAACCCCTGCCGGCACC

L44-Z_Europe (n=22) GTGCAGCCCCTACTTCCAGTCGCTGTTCATGGACAACCCCTGCCGGCACC

E-strain_Europe lab (n=59) GTGCAGCCCCTACTTCCAGTCGCTGTTCATGGACAACCCCTGCCGGCACC

E-strain_America lab (n=21) GTGCAGCCCCTACTTCCAGTCGCTGTTCATGGACAACCCCTGCCGGCACC

E-strain_America field (n=24) GTGCAGCCCCTACTTCCAGTCGCTGTTCATGGACAACCCCTGCCGGCACC

L195_Europe (n=22) GTGCAGCCCCTACTTCCAGTCGCTGTTCATGGACAACCCCTGCCGGCACC

L173__Europe (n=20) GTGCAGCCCCTACTTCCAGTCGCTGTTCATGGACAACCCCTGCCGGCACC

L205_Europe (n=36) GTGCAGCCCCTACTTCCAGTCGCTGTTCATGGACAACCCCTGCCGGCACC

L215_Europe (n=19) GTGCAGCCCCTACTTCCAGTCGCTGTTCATGGACAACCCCTGCCGGCACC

L44-E_Europe (n=28) GTGCAGCCCCTACTTCCAGTCGCTGTTCATGGACAACCCCTGCCGGCACC

260 270 280 290 300

....|....|....|....|....|....|....|....|....|....|

Z-strain_Europe lab (n=52) CGATCATAATAATGCGCGACATAAAGTACTGCGACTTGAAGGCGGTCGTG

Z-strain_America lab (n=29) CGATCATAATAATGCGCGACATAAAGTACTGCGACTTGAAGGCGGTCGTG

Z-strain_America field (n=27) CGATCATAATAATGCGCGACATAAAGTACTGCGACTTGAAGGCGGTCGTG

L165_Europe (n=23) CGATCATAATAATGCGCGACATAAAGTACTGCGACTTGAAGGCGGTCGTG

L185_Europe (n=20) CGATCATAATAATGCGCGACATAAAGTACTGCGACTTGAAGGCGGTCGTG

L220_Europe (n=10) CGATCATAATAATGCGCGACATAAAGTACTGCGACTTGAAGGCGGTCGTG

L237_Europe (n=14) CGATCATAATAATGCGCGACATAAAGTACTGCGACTTGAAGGCGGTCGTG

L44-Z_Europe (n=22) CGATCATAATAATGCGCGACATAAAGTACTGCGACTTGAAGGCGGTCGTG

E-strain_Europe lab (n=59) CGATCATAATAATGCGCGACATAAAGTACTGCGACTTGAAGGCGGTCGTG

E-strain_America lab (n=21) CGAT**M**ATAATAATGCGCGACATAAAGTACTGCGACTTGAAGGCGGTCGTG

E-strain_America field (n=24) CGAT**M**ATAATAATGCGCGACATAAAGTACTGCGACTTGAAGGCGGTCGTG

L195_Europe (n=22) CGATCATAATAATGCGCGACATAAAGTACTGCGACTTGAAGGCGGTCGTG

L173__Europe (n=20) CGATCATAATAATGCGCGACATAAAGTACTGCGACTTGAAGGCGGTCGTG

L205_Europe (n=36) CGATCATAATAATGCGCGACATAAAGTACTGCGACTTGAAGGCGGTCGTG

L215_Europe (n=19) CGATCATAATAATGCGCGACATAAAGTACTGCGACTTGAAGGCGGTCGTG

L44-E_Europe (n=28) CGATCATAATAATGCGCGACATAAAGTACTGCGACTTGAAGGCGGTCGTG

310 320 330 340 350

....|....|....|....|....|....|....|....|....|....|

Z-strain_Europe lab (n=52) GACTTCATGTACCGCGGGGAGATAAACGTGTCGCAGGACCAGATCTCGGC

Z-strain_America lab (n=29) GACTTCATGTACCGCGGGGAGATAAACGTGTCGCAGGACCAGATCTCGGC

Z-strain_America field (n=27) GACTTCATGTACCGCGGGGAGATAAACGTGTCGCAGGACCAGATCTCGGC

L165_Europe (n=23) GACTTCATGTACCGCGGGGAGATAAACGTGTCGCAGGACCAGATCTCGGC

L185_Europe (n=20) GACTTCATGTACCGCGGGGAGATAAACGTGTCGCAGGACCAGATCTCGGC

L220_Europe (n=10) GACTTCATGTACCGCGGGGAGATAAACGTGTCGCAGGACCAGATCTCGGC

L237_Europe (n=14) GACTTCATGTACCGCGGGGAGATAAACGTGTCGCAGGACCAGATCTCGGC

L44-Z_Europe (n=22) GACTTCATGTACCGCGGGGAGATAAACGTGTCGCAGGACCAGATCTCGGC

E-strain_Europe lab (n=59) GACTTCATGTACCGCGGGGAGATAAACGTGTCGCAGGA**Y**CAGATCTCGGC

E-strain_America lab (n=21) GACTTCATGTACCGCGGGGAGATAAACGTGTCGCAGGACCAGATCTCGGC

E-strain_America field (n=24) GACTTCATGTACCGCGGGGAGATAAACGTGTCGCAGGACCAGATCTCGGC

L195_Europe (n=22) GACTTCATGTACCGCGGGGAGATAAACGTGTCGCAGGACCAGATCTCGGC

L173__Europe (n=20) GACTTCATGTACCGCGGGGAGATAAACGTGTCGCAGGACCAGATCTCGGC

L205_Europe (n=36) GACTTCATGTACCGCGGGGAGATAAACGTGTCGCAGGACCAGATCTCGGC

L215_Europe (n=19) GACTTCATGTACCGCGGGGAGATAAACGTGTCGCAGGA**T**CAGATCTCGGC

L44-E_Europe (n=28) GACTTCATGTACCGCGGGGAGATAAACGTGTCGCAGGACCAGATCTCGGC

360 370 380 390 400

....|....|....|....|....|....|....|....|....|....|

Z-strain_Europe lab (n=52) GCTGCTGAAGGTGGCGGAGACGCTGAAGATCAGGGGCCTGAC**S**GA**Y**GTGA

Z-strain_America lab (n=29) GCTGCTGAAGGTGGCGGAGACGCTGAAGATCAGGGGCCTGAC**G**GACGTGA

Z-strain_America field (n=27) GCTGCTGAAGGTGGCGGAGACGCTGAAGATCAGGGGCCTGAC**G**GA**Y**GTGA

L165_Europe (n=23) GCTGCTGAAGGTGGCGGAGACGCTGAAGATCAGGGGCCTGACCGACGTGA

L185_Europe (n=20) GCTGCTGAAGGTGGCGGAGACGCTGAAGATCAGGGGCCTGACCGACGTGA

L220_Europe (n=10) GCTGCTGAAGGTGGCGGAGACGCTGAAGATCAGGGGCCTGAC**G**GACGTGA

L237_Europe (n=14) GCTGCTGAAGGTGGCGGAGACGCTGAAGATCAGGGGCCTGACCGACGTGA

L44-Z_Europe (n=22) GCTGCTGAAGGTGGCGGAGACGCTGAAGATCAGGGGCCTGACCGACGTGA

E-strain_Europe lab (n=59) GCTGCTGAAGGTGGC**S**GAGACGCTGAAGATCAGGGGCCTGAC**S**GACGTGA

E-strain_America lab (n=21) GCTGCTGAAGGTGGC**C**GAGACGCTGAAGATCAGGGGCCTGAC**S**GACGTGA

E-strain_America field (n=24) GCTGCTGAAGGTGGC**C**GAGACGCTGAAGATCAGGGGCCTGAC**S**GACGTGA

L195_Europe (n=22) GCTGCTGAAGGTGGC**S**GAGACGCTGAAGATCAGGGGCCTGACCGACGTGA

L173__Europe (n=20) GCTGCTGAAGGTGGCGGAGACGCTGAAGATCAGGGGCCTGACCGACGTGA

L205_Europe (n=36) GCTGCTGAAGGTGGCGGAGACGCTGAAGATCAGGGGCCTGACCGACGTGA

L215_Europe (n=19) GCTGCTGAAGGTGGCGGAGACGCTGAAGATCAGGGGCCTGAC**G**GACGTGA

L44-E_Europe (n=28) GCTGCTGAAGGTGGC**C**GAGACGCTGAAGATCAGGGGCCTGACCGACGTGA

410 420 430 440 450

....|....|....|....|....|....|....|....|....|....|

Z-strain_Europe lab (n=52) GCGGCGAGCAGGCCGTGCTGCGGCCGGGCGGCGTGGAGCGCGGGTCCAAG

Z-strain_America lab (n=29) GCGGCGAGCAGGCCGTGCTGCGGCCGGGCGGCGTGGAGCGCGG**C**TCCAAG

Z-strain_America field (n=27) GCGGCGAGCAGGCCGTGCTGCGGCCGGGCGGCGTGGAGCGCGGGTCCAAG

L165_Europe (n=23) GCGGCGAGCAGGCCGTGCTGCGGCCGGGCGGCGTGGAGCGCGGGTCCAAG

L185_Europe (n=20) GCGGCGAGCAGGCCGTGCTGCGGCCGGGCGGCGTGGAGCGCGGGTCCAAG

L220_Europe (n=10) GCGGCGAGCAGGCCGTGCTGCGGCCGGGCGGCGTGGAGCGCGG**S**TCCAAG

L237_Europe (n=14) GCGGCGAGCAGGCCGTGCTGCGGCCGGGCGGCGTGGAGCGCGGGTCCAAG

L44-Z_Europe (n=22) GCGGCGAGCAGGCCGTGCTGCGGCCGGGCGGCGTGGAGCGCGGGTCCAAG

E-strain_Europe lab (n=59) GCGG**Y**GAGCAGGCCGTGCTGCGGCCGGGCGGCGTGGAGCG**G**GGGTCCAAG

E-strain_America lab (n=21) GCGGCGAGCAGGCCGTGCTGCGGCCGGGCGGCGTGGAGCGCGGGTCCAAG

E-strain_America field (n=24) GCGGCGAGCAGGCCGTGCTGCGGCCGGGCGGCGTGGAGCGCGGGTCCAAG

L195_Europe (n=22) GCGGCGAGCAGGCCGTGCTGCGGCCGGGCGGCGTGGAGCGCGGGTCCAAG

L173__Europe (n=20) GCGGCGAGCAGGCCGTGCTGCGGCCGGGCGGCGTGGAGCGCGGGTCCAAG

L205_Europe (n=36) GCGGCGAGCAGGCCGTGCTGCGGCCGGGCGGCGTGGAGCGCGGGTCCAAG

L215_Europe (n=19) GCGGCGAGCAGGCCGTGCTGCGGCCGGGCGGCGTGGAGCGCGGGTCCAAG

L44-E_Europe (n=28) GCGG**T**GAGCAGGCCGTGCTGCGGCCGGGCGGCGTGGAGCG**G**GGGTCCAAG

460 470 480 490 500

....|....|....|....|....|....|....|....|....|....|

Z-strain_Europe lab (n=52) CGGCCGCAGAGCCGCGAGCCCGCCTCGCCGGCGAAGGCGCGGCGCCGCTC

Z-strain_America lab (n=29) CGGCCGCAGAGCCGCGAGCCCGCCTCGCCGGCGAAGGCGCGGCGCCGCTC

Z-strain_America field (n=27) CGGCCGCAGAGCCGCGAGCCCGCCTCGCCGGCGAAGGCGCGGCGCCGCTC

L165_Europe (n=23) CGGCCGCAGAGCCGCGAGCCCGCCTCGCCGGCGAAGGCGCGGCGCCGCTC

L185_Europe (n=20) CGGCCGCAGAGCCGCGAGCCCGCCTCGCCGGCGAAGGCGCGGCGCCGCTC

L220_Europe (n=10) CGGCCGCAGAGCCGCGAGCCCGCCTCGCCGGCGAAGGCGCGGCGCCGCTC

L237_Europe (n=14) CGGCCGCAGAGCCGCGAGCCCGCCTCGCCGGCGAAGGCGCGGCGCCGCTC

L44-Z_Europe (n=22) CGGCCGCAGAGCCGCGAGCCCGCCTCGCCGGCGAAGGCGCGGCGCCGCTC

E-strain_Europe lab (n=59) CGGCCGCAGAGCCGCGAGCCCGCCTCGCCGGCGAAGGCGCGGCGCCGCTC

E-strain_America lab (n=21) CGGCCGCAGAGCCGCGAGCCCG**A**CTCGCCGGCGAAGGCGCGGCGCCGCTC

E-strain_America field (n=24) CGGCCGCAGAGCCGCGAGCCCG**A**CTCGCCGGCGAAGGCGCGGCGCCGCTC

L195_Europe (n=22) CGGCCGCAGAGCCGCGAGCCCG**MS**TCGCCGGCGAAGGCGCGGCGCCGCTC

L173__Europe (n=20) CGGCCGCAGAGCCGCGAGCCCGCCTCGCCGGCGAAGGCGCGGCGCCGCTC

L205_Europe (n=36) CGGCCGCAGAGCCGCGAGCCCGCCTCGCCGGCGAAGGCGCGGCGCCGCTC

L215_Europe (n=19) CGGCCGCAGAGCCGCGAGCCCGCCTCGCCGGCGAAGGCGCGGCGCCGCTC

L44-E_Europe (n=28) CGGCCGCAGAGCCGCGAGCCCGCCTCGCCGGCGAAGGCGCGGCGCCGCTC

510 520 530 540 550

....|....|....|....|....|....|....|....|....|....|

Z-strain_Europe lab (n=52) GGGCGAGCGCAGCCCGGGCCCGAGCCCGCGCCGCTCGCCCAGCACGCCCA

Z-strain_America lab (n=29) GGGCGAGCGCAGCCCGGGCCCGAGCCCGCGCCGCTCGCCCAGCACGCCCA

Z-strain_America field (n=27) GGGCGAGCGCAGCCCGGGCCCGAGCCCGCGCCGCTCGCCCAGCACGCCCA

L165_Europe (n=23) GGGCGAGCGCAGCCCGGGCCCGAGCCCGCGCCGCTCGCCCAGCACGCCCA

L185_Europe (n=20) GGGCGAGCGCAGCCCGGGCCCGAGCCCGCGCCGCTCGCCCAGCACGCCCA

L220_Europe (n=10) GGGCGAGCGCAGCCCGGGCCCGAGCCCGCGCCGCTCGCCCAGCACGCCCA

L237_Europe (n=14) GGGCGAGCGCAGCCCGGGCCCGAGCCCGCGCCGCTCGCCCAGCACGCCCA

L44-Z_Europe (n=22) GGGCGAGCGCAGCCCGGGCCCGAGCCCGCGCCGCTCGCCCAGCACGCCCA

E-strain_Europe lab (n=59) GGGCGAGCGCAGCCCGGGCCCGAGCCCGCGCCGCTCGCCCAGCACGCCCA

E-strain_America lab (n=21) GGGCGAGCGCAGCCCGGGCCCGAGCCCGCGCCGCTCGCCCAGCACGCCCA

E-strain_America field (n=24) GGGCGAGCGCAGCCCGGGCCCGAGCCCGCGCCGCTCGCCCAGCACGCCCA

L195_Europe (n=22) GGGCGAGCGCAGCCCGGGCCCGAGCCCGCGCCGCTCGCCCAGCACGCCCA

L173__Europe (n=20) GGGCGAGCGCAGCCCGGGCCCGAGCCCGCGCCGCTCGCCCAGCACGCCCA

L205_Europe (n=36) GGGCGAGCGCAGCCCGGGCCCGAGCCCGCGCCGCTCGCCCAGCACGCCCA

L215_Europe (n=19) GGGCGAGCGCAGCCCGGGCCCGAGCCCGCGCCGCTCGCCCAGCACGCCCA

L44-E_Europe (n=28) GGGCGAGCGCAGCCCGGGCCCGAGCCCGCGCCGCTCGCCCAGCACGCCCA

560 570 580 590 600

....|....|....|....|....|....|....|....|....|....|

Z-strain_Europe lab (n=52) GCGCGCCGCCGGAGCTGCCGCCGGACGCCGCGCACGCCGCGCCCGCCGGC

Z-strain_America lab (n=29) GCGCGCCGCCGGAGCTGCCGCCGGACGCCGCGCACGCCGCGCCCGCCGGC

Z-strain_America field (n=27) GCGCGCCGCCGGAGCTGCCGCCGGACGCCGCGCACGCCGCGCCCGCCGGC

L165_Europe (n=23) GCGCGCCGCCGGAGCTGCCGCCGGACGCCGCGCACGCCGCGCCCGCCGGC

L185_Europe (n=20) GCGCGCCGCCGGAGCTGCCGCCGGACGCCGCGCACGCCGCGCCCGCCGGC

L220_Europe (n=10) GCGCGCCGCCGGAGCTGCCGCCGGACGCCGCGCACGCCGCGCCCGCCGGC

L237_Europe (n=14) GCGCGCCGCCGGAGCTGCCGCCGGACGCCGCGCACGCCGCGCCCGCCGGC

L44-Z_Europe (n=22) GCGCGCCGCCGGAGCTGCCGCCGGACGCCGCGCACGCCGCGCCCGCCGGC

E-strain_Europe lab (n=59) GCGCGCCGCCGGAGCTGCCGCCGGACGCCGCGCACGCCGCGCCCGCCGGC

E-strain_America lab (n=21) GCGCGCCGCCGGAGCTGCCGCCGGACGCCGCGCACGCCGCGCCCGCCGGC

E-strain_America field (n=24) GCGCGCCGCCGGAGCTGCCGCCGGACGCCGCGCACGCCGCGCCCGCCGGC

L195_Europe (n=22) GCGCGCCGCCGGAGCTGCCGCCGGACGCCGCGCACGCCGCGCCCGCCGGC

L173__Europe (n=20) GCGCGCCGCCGGAGCTGCCGCCGGACGCCGCGCACGCCGCGCCCGCCGGC

L205_Europe (n=36) GCGCGCCGCCGGAGCTGCCGCCGGACGCCGCGCACGCCGCGCCCGCCGGC

L215_Europe (n=19) GCGCGCCGCCGGAGCTGCCGCCGGACGCCGCGCACGCCGCGCCCGCCGGC

L44-E_Europe (n=28) GCGCGCCGCCGGAGCTGCCGCCGGACGCCGCGCACGCCGCGCCCGCCGGC

610 620 630 640 650

....|....|....|....|....|....|....|....|....|....|

Z-strain_Europe lab (n=52) CCGCCGCCGCACGCCGACGACGTCGACATCCGCCCCGGCATCGC**M**GAGAT

Z-strain_America lab (n=29) CCGCCGC**T**GCACGCCGACGACGTCGACATCCGCCCCGGCATCGCCGAGAT

Z-strain_America field (n=27) CCGCCGCCGCACGCCGACGACGTCGACATCCGCCCCGGCATCGCCGAGAT

L165_Europe (n=23) CCGCCGCCGCACGCCGACGACGTCGACATCCGCCCCGGCATCGC**A**GAGAT

L185_Europe (n=20) CCGCCGCCGCACGCCGACGACGTCGACATCCGCCCCGGCATCGC**A**GAGAT

L220_Europe (n=10) CCGCCGCCGCACGCCGACGACGTCGACATCCGCCCCGGCATCGC**A**GAGAT

L237_Europe (n=14) CCGCCGCCGCACGCCGACGACGTCGACATCCGCCCCGGCATCGC**A**GAGAT

L44-Z_Europe (n=22) CCGCCGCCGCACGCCGACGACGTCGACATCCGCCCCGGCATCGC**A**GAGAT

E-strain_Europe lab (n=59) CCGCCGC**T**GCACGCCGACGACGTCGACATCCGCCCCGGCATCGCCGAGAT

E-strain_America lab (n=21) CCGCCGC**T**GCACGCCGACGACGTCGACATCCGCCCCGGCATCGCCGAGAT

E-strain_America field (n=24) CCGCCGC**T**GCACGCCGACGACGTCGACATCCGCCCCGGCATCGCCGAGAT

L195_Europe (n=22) CCGCCGC**T**GCACGCCGACGACGTCGACATCCGCCCCGGCATCGCCGAGAT

L173__Europe (n=20) CCGCCGC**T**GCACGCCGACGACGTCGACATCCGCCCCGGCATCGCCGAGAT

L205_Europe (n=36) CCGCCGC**T**GCACGCCGACGACGTCGACATCCGCCCCGGCATCGCCGAGAT

L215_Europe (n=19) CCGCCGC**T**GCACGCCGACGACGTCGACATCCGCCCCGGCATCGCCGAGAT

L44-E_Europe (n=28) CCGCCGC**T**GCACGCCGACGACGTCGACATCCGCCCCGGCATCGCCGAGAT

660

....|....|....|....

Z-strain_Europe lab (n=52) GATCCGCGAGGAGGAGAGG

Z-strain_America lab (n=29) GATCCGCGAGGAGGAGAGG

Z-strain_America field (n=27) GATCCGCGAGGAGGAGAGG

L165_Europe (n=23) GATCCGCGAGGAGGAGAGG

L185_Europe (n=20) GATCCGCGAGGAGGAGAGG

L220_Europe (n=10) GATCCGCGAGGAGGAGAGG

L237_Europe (n=14) GATCCGCGAGGAGGAGAGG

L44-Z_Europe (n=22) GATCCGCGAGGAGGAGAGG

E-strain_Europe lab (n=59) GATCCGCGAGGAGGAGAGG

E-strain_America lab (n=21) GATCCGCGAGGAGGAGAGG

E-strain_America field (n=24) GATCCGCGAGGAGGAGAGG

L195_Europe (n=22) GATCCGCGAGGAGGAGAGG

L173__Europe (n=20) GATCCGCGAGGAGGAGAGG

L205_Europe (n=36) GATCCGCGAGGAGGAGAGG

L215_Europe (n=19) GATCCGCGAGGAGGAGAGG

L44-E_Europe (n=28) GATCCGCGAGGAGGAGAGG
